# A Deeply Branching Thermophilic Bacterium with an Ancient Acetyl-CoA Pathway Dominates a Subsurface Ecosystem

**DOI:** 10.1371/journal.pone.0030559

**Published:** 2012-01-27

**Authors:** Hideto Takami, Hideki Noguchi, Yoshihiro Takaki, Ikuo Uchiyama, Atsushi Toyoda, Shinro Nishi, Gab-Joo Chee, Wataru Arai, Takuro Nunoura, Takehiko Itoh, Masahira Hattori, Ken Takai

**Affiliations:** 1 Microbial Genome Research Group, Japan Agency for Marine-Earth Science and Technology (JAMSTEC), Yokosuka, Japan; 2 Department of Biological Sciences, Tokyo Institute of Technology, Yokohama, Japan; 3 Laboratory of Genome Informatics, National Institute for Basic Biology, National Institutes of Natural Sciences, Aichi, Japan; 4 National Institute of Genetics, Mishima, Japan; 5 Subground Animalcule Retrieval (Sugar) Project, Natsushima, Japan; 6 Center for Omics and Bioinformatics, Graduate School of Frontier Sciences, The University of Tokyo, Kashiwa, Japan; Argonne National Laboratory, United States of America

## Abstract

A nearly complete genome sequence of *Candidatus* ‘Acetothermum autotrophicum’, a presently uncultivated bacterium in candidate division OP1, was revealed by metagenomic analysis of a subsurface thermophilic microbial mat community. Phylogenetic analysis based on the concatenated sequences of proteins common among 367 prokaryotes suggests that *Ca.* ‘A. autotrophicum’ is one of the earliest diverging bacterial lineages. It possesses a folate-dependent Wood-Ljungdahl (acetyl-CoA) pathway of CO_2_ fixation, is predicted to have an acetogenic lifestyle, and possesses the newly discovered archaeal-autotrophic type of bifunctional fructose 1,6-bisphosphate aldolase/phosphatase. A phylogenetic analysis of the core gene cluster of the acethyl-CoA pathway, shared by acetogens, methanogens, some sulfur- and iron-reducers and dechlorinators, supports the hypothesis that the core gene cluster of *Ca.* ‘A. autotrophicum’ is a particularly ancient bacterial pathway. The habitat, physiology and phylogenetic position of *Ca.* ‘A. autotrophicum’ support the view that the first bacterial and archaeal lineages were H_2_-dependent acetogens and methanogenes living in hydrothermal environments.

## Introduction

Because most deeply branching bacteria are thermophiles, the hypothesis that bacteria arose from a thermophilic ancestor is widely but not universally accepted [1], [2]. Since the discovery of deep-sea hydrothermal systems more than 30 years ago, this hypothesis has also been supported by the geological and geochemical outlines of early earth environments hosting ancient life [3], [4]. Initially, all types of deep-sea hydrothermal systems were considered possible cradles for early life; however, recently, specific types have been proposed to be the most plausible places, including low-temperature alkaline H_2_-rich hydrothermal vents such as the Lost City hydrothermal field discovered near the Mid-Atlantic Ridge [5], [6], high-temperature H_2_-rich black smoker vents such as the Kairei hydrothermal field in the Indian Ocean [7], [8], and highly alkaline white smoker vents in the Hadean and the early Archean ocean [9]. In the pioneering studies of Wächthershäuser, the theory of surface metabolism in the early evolution of life was formulated [10], and recently a model for prebiotic cellular and biochemical evolution in an alkaline hydrothermal vent chimney has been proposed with an evolutionary scenario of the acetyl-CoA pathway of CO_2_ fixation and central intermediary metabolism leading to the synthesis of the constituents of purines and pyrimidines [11], [12]. Moreover, acetogenesis and methanogenesis enabled by this pathway have been proposed to be the ancestral forms of energy metabolism in the first free-living bacterial and archaeal ancestors [11]. A prediction of that view is that the acetyl-CoA pathway should be found in deeply branching bacterial lineages.

Candidate division OP1 was first characterized in a culture-independent molecular phylogenetic survey based on the 16S rRNA gene of the Obsidian Pool, a 75 to 95°C hot spring at the northern flank of the Yellowstone caldera, and it was considered a thermophile [13]. This phylotype has been detected in several deep-sea hydrothermal environments [14]–[16] and geothermal waters such as Icelandic alkaline geothermal water and hot springs in the northwestern Great Basin [17], [18]. In addition, OP1 phylotype has been detected in a microbial mat community present in a 70°C hot water stream with a weakly acidic pH 5.1 and a low oxidation-reduction potential value (−130 mV) in a Japanese epithermal mine, the bacterial community we investigated in a previous study [19], [20]. Although this hot water stream is poor in organic compounds having two or more carbon atoms, plenty of geological energy and carbon sources, such as hydrogen, carbon dioxide, methane, sulfide and ammonium, are supplied by the geothermal aquifer [19], [20]. These environmental settings where OP1 phylotypes were detected seem to fit the above prebiotic evolutionary scenario and motivated us to investigate the microbial mat community containing OP1 phylotype through metagenomic analysis in search of evidence that might link this lineage and setting to the earliest phases of evolution.

To elucidate the physiology and genomic traits of the predominant archaeal species in the microbial mat community, a metagenomic fosmid library has been constructed in the previous study [21] and then the genome of *Candidatus* ‘Caldiarchaeum subterraneum’ within hot water crenarchaeotic group I (HWCGI) has been reconstructed by the metagenomic approach [22]. Here, we assembled genomic information of the OP1 phylotype *Candidatus* ‘Acetothermum autotrophicum’, by using the same metagenomic library, identified a deeply branching thermophilic bacterium with a deeply diverging acetyl-CoA pathway.

## Results

### Reconstruction of Genomic Fragments Derived from the OP1 phylotype

**Figure 1:**
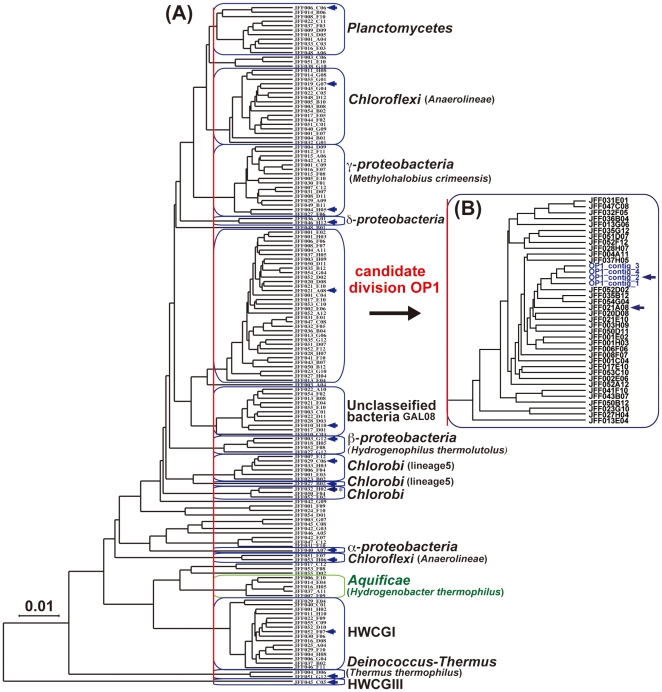
Hierarchical clustering pattern based on codon usages of the genes. The fosmid clones within 0.04 of the Euclidean distances corresponding to the red line are evaluated as the same species groups. Blue box indicates a fosmid group derived from one species and blue arrow shows the clone containing the 16S rRNA gene. Asterisk shows the fosmid clone containing partial 16S rDNA sequence (631 bp). Blue characters show *Ca.* ‘A. autotrophicum’ (Candidate division OP1) contigs. The group categorized into *Aquificae* (a green box) was characterized on the basis of significant sequence similarities of the genes identified in the fosmid clones to those of *Hydrogenobacter thermophilus*. A) Relationship between each fosmid based on the hierarchical clustering. B) Relationship between the fosmids and contigs in the cluster of *Ca.* ‘A. autotrophicum’.

**Figure 2:**
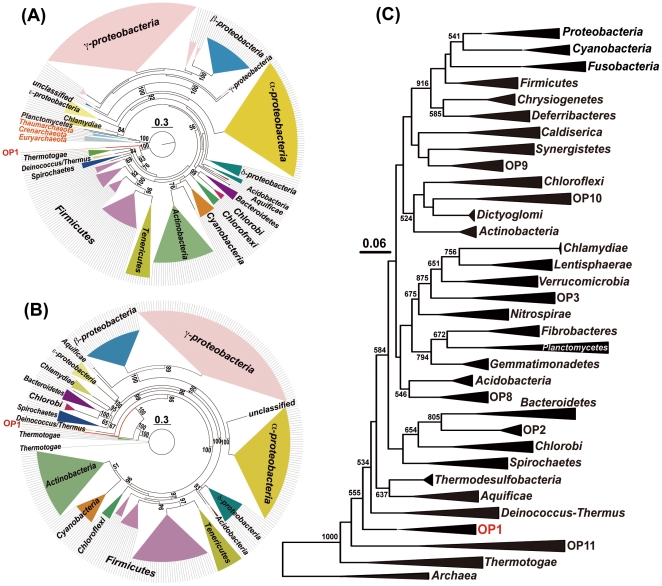
Phylogenetic position of *Ca.* ‘A. autotrophicum’. A maximum likelihood tree was constructed by using RAxML [51] and PHYML [49]. A) A concatenated alignment of the sequences of 4 common proteins (Pgk, PyrG, rplK, and rpsI) among 358 bacteria, including *Ca.* ‘A. autotrophicum’ and nine archaea. B) A concatenated alignment of the sequences of 16 common protein sequences (DnaG, Frr, InfC, NusA, pgK, PyrG, RplA, RplK, RplL, RplS, RplT, RpmA, RpoB, RpsB, RpsI, and SmpB) among 358 bacteria including *A. autotrophicum*. C) Phylogenetic position of candidate division OP1 containing *A. autotrophicum* among the prokaryotic major lineages (phyla) based on 16S rRNA genes. The name of cultured and uncultured species used for construction of the phylogenetic tree is listed in Table S2 and S3. The numbers indicate the percentages of bootstrap support. Bootstrap values less than 50% were omitted from this figure.

We detected in total 41 positive clones possessing 16S rRNA genes by dot blot hybridization from 3,375 fosmid clones. All sequences of the 16S rRNA genes were determined and classified into 15 groups based on sequence similarity as shown in Table 1. We sequenced 151 fosmid clones including 136 randomly selected clones and 15 representative clones carrying the 16S rRNA gene. Except for the shortest clone (JFF013_E04) with only 1,685 codons, the internal codon numbers ranged from 7,996 to 14,272. Using the codon usage pattern in each clone as a dataset, a hierarchical clustering analysis was performed (Figure 1A). When we set the clustering cutoff distance at 0.04 determined from a test using a simulated dataset (Figure S2A), the 151 sequenced clones were classified into 28 species groups, among which the most major group was OP1 phylotype group consisting of 34 fosmid clones. The 16S rDNA sequences of the OP1 species identified in the 4 fosmid clones are identical to each other with one- or two-base substitution in the comparison of whole region (>99.9%-identity). The representative sequence of OP1 phylotype (JFF021_A08) formed a clade with the previously known OP1 phylotypes (Figure 2C).

**Table 1:** Phylotypes based on 16S rDNA sequences detected in metagenomic fosmid library.

phylotype	Clone No. used for phylogenetic analysis*	Number of clones (Identity %)
Candidate division OP1	JFF021_A08	4 (99.9)
*Chloroflexi-Anaerolineae*_1	JFF019_G07	2 (100)
Chloroflexi-Anaerolineae_2	JFF053_H06	1
Chlorobi-lineage 5_1	JFF029_C06	1
Chlorobi-lineage 5-2	JFF027_B02	1
Chlorobi**	JFF032_H02	1
*Planctomyces*	JFF006_C06	1
*Alphaproteobacteria*	JFF040_A07	1
*Betaproteobacteria* 1)	JFF003_G12	1
*Gammaproteobacteria* 2)	JFF004_H05	2 (100)
*Deltaproteobacteria*	JFF046_H12	2 (99.9)
Unclassified bacteria-GAL08	JFF010_H10	3 (99.9)
*Deinococcus-Thermus* 3)	JFF051_G12	1
HWCGI 4)	JFF052_F07	18 (99.9)
HWCGIII 5)	JFF045_C05	2 (100)

*16S rDNA sequence identified in each representative clone was used for phylogenetic analysis in Figure S6.

**partial sequence. Numbers in the parenthesis show the identuty % among the same phylotypes.

1) *Hydrogenophilus thermoluteolus*,

2) *Methylohalobius crimeensis*,

3) *Thermus* sp. TH92,

4) *Ca.* ‘Caldiarchaeum subterraneum’,

5) *Ca.* ‘Nitrosocaldus’ sp.

We gathered more genomic information of the OP1 species from the rest of fosmid clones based on the codon usage of their end sequences and successfully assembled four contigs from the sequenced 34 fosmid clones and additional pyrosequencing reads from 143 clones out of the 176 clones predicted to be a part of the genome of OP1 phylotype. To confirm the homogeneity among the assembled contigs, we added 4 assembled contigs to the codon-usage-based clustering analysis in Figure 1A and found that they were more closely related to each other than to any other individual clone of the OP1 phylotype (Figure 1B), indicating that these contigs are homogeneous enough to be considered as part of the same species' genome. Here, we designated this OP1 phylotype, which is a host bacterium of the composite genome as *Candidatus* ‘Acetothermus autotrophicum’ (see discussion for description of *Ca.* ‘A. autotrophicum’). The four contigs consisting of 1,970,005 bp in total from *Ca.* ‘A. autotrophicum’ contain 1,982 predicted protein coding genes, 46 tRNA and 3 rRNA genes (Table 2). These genes were found to cover approximately 73% out of the common 306 genes shared among >95% of all the sequenced bacteria whose genome sizes are larger than 1.5 Mbp (Table S1). However, the set of missing common genes contained a large ribosomal cluster that is likely to be located on the same fosmid clone. The coverage was slightly increased up to 79% if genes that are physically clustered in at least 90% of the sequenced genomes were counted as one cluster (Table S1).

**Table 2:** General feature of the composite genome of *Candidatus* ‘A. autotrophicum’.

General features	Contig 1	Contig 2	Contig 3	Contig 4
Size (bp)	143,068	391.365	518,087	917,485
G+C content (%)	57.8	57.7	58.0	58.0
Protein coding genes	146	394	519	921
Function assigned	67	225	302	573
Hypothetical conserved	34	69	92	167
Hypothetical	45	100	125	181
rRNA genes	0	3	0	0
tRNA genes	7	6	11	19

### *Ca.* ‘Acetothermus autotrophicum’ that is Placed in the Deeply Branching Bacterial Lineage

We conducted a genome-wide phylogenetic analysis using a concatenated alignment of broadly conserved protein-coding genes [23]. A phylogenetic tree based on the concatenated alignment of four genes broadly conserved in all 367 prokaryotic species (Table S2), including nine archaeal species, demonstrated that *Ca.* ‘A. autotrophicum’ is the earliest species to have diverged from the common ancestral lineage among 358 bacteria; this result was also supported by its higher bootstrap value near the root (Figure 2A). We constructed another phylogenetic tree based on the concatenated alignment of 16 broadly conserved genes among 358 bacteria, and it shows topology similar to that of the former tree (Figure 2B). In particular, the phylogenetic position of *Ca.* ‘A. autotrophicum’ was also reproduced in this tree. Although the topologies of the subtrees containing these phyla were slightly different between the two phylogenetic trees, the phylogenetic tree based on the 16S rDNA sequence also supported that the phylogenetic position of *Ca.* ‘A. autotrophicum’ identified in this study is near *Deinococcus-Thermus* and *Thermotogae* (Figure 2C).

The G+C contents of the 16S rRNA gene of *Ca.* ‘A. autotrophicum’ was 61.9%. It is known that the G+C contents of 16S rRNA genes from thermophiles and hyperthermophiles are generally higher than those from mesophiles and psychrophiles, and that a linear correlation between G+C content and optimal temperature for growth has been observed [24]. We plotted the maximum temperature for growth of 10 prokaryotic species against the G+C contents of their 16S rRNA genes. As shown in Figure S2, a clear correlation between the two parameters was confirmed. We then estimated the maximum temperature for growth of *Ca.* ‘A. autotrophicum’ to be 84.7°C according to a regression equation. Thus, *Ca.* ‘A. autotrophicum’ appears to be a thermophilic deeply branching bacterium.

### Genes for Acetyl-CoA Pathway Identified in *Ca.* ‘A. autotrophicum’

Metabolism of *Ca.* ‘A. autotrophicum’ as reconstructed by metagenomic analysis revealed its ability to generate acetyl-CoA serves as biosynthetic starting material from CO_2_ and H_2_ through the acetyl-CoA pathway (Figure S3A). In fact, *Ca.* ‘A. autotrophicum’ was found to possess all genes (OP3C167–168, 178, 183–184, 187–188, and OP2C251) related to acetyl-CoA pathway to generate acetyl-CoA leading to acetogenesis concomitant with ATP synthesis although the genes for generation of acetate, phosphate transacetylase and acetate kinase, identified in the modern acetogens are missing in the composite genome of *Ca.* ‘A. autotrophicum’. These genes could be hidden in the contig gaps. One CO_2_ molecule is reduced to a cofactor-bound methyl group, formally through three hydride (H^−^) transfers in the eastern branch of this pathway. The methyl group is donated to the acetyl-CoA decarbonylase/synthase (ACDS) complex, shared by acetogens and methanogens, to synthesize acetyl-CoA and to generate a reduced CO molecule from an additional CO_2_ molecule. Both acetogens and methanogens synthesise ATP and acetyl-CoA with the help of the acetyl-CoA pathway [11], [25]. The genes encoding the ACDS complex that comprise the core enzymes of the acetyl-CoA pathway are located in an operon-like cluster and are well conserved in acetogens and methanogens (Figure S3B). This *acs/cdh* (*acs* in bacteria and *cdh* in archaea) gene cluster is also conserved in some sulfur- and iron-reducers, as well as in dechlorinators classified into the limited taxa. In these gene clusters, five genes (orthologous groups) including *acsA-D/cdhA-D* and *acsF/cdhF* are conserved in both bacteria and archaea.

**Figure 3:**
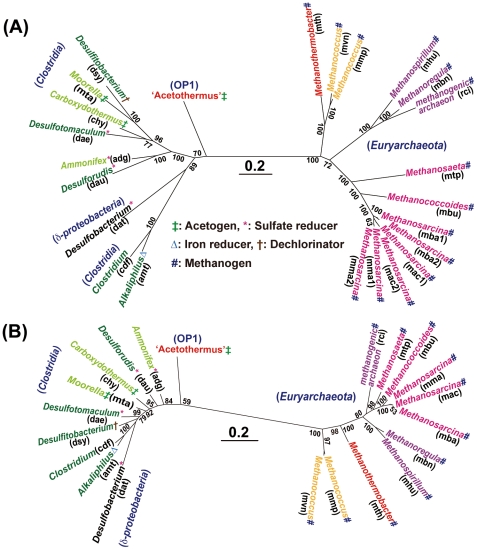
Phylogenetic trees based on the ACDS complex and the 16S rRNA genes. A) Concatenated alignment of the sequences of five proteins encoded by the core genes (*acs/cdhA-D* and *acs/cdhF*) involved in the ACDS complex in the acetyl-CoA pathway. B) Alignment of the sequences of 16S rRNA genes. The complete species names for all abbreviations are listed in the legend of Figure S3. The numbers indicate the percentage of bootstrap samples supporting the internal branches. Bootstrap values less than 50% were omitted from this figure. Dark green, *Thermoanaerobacterales*; light green, *Clostridiales*; black, *Desulfobacterales*; red, *Methanobacteriales*; orange, *Methanococcales*; dark blue, *Methanomicrobiales*; magenta, *Methanosarcinales*.

**A phylogenetic analysis using the concatenated alignment of the five conserved** proteins comprising the ACDS complex in nine bacteria (including *Ca.* ‘A. autotrophicum’) and 11 archaea indicated that a large archaeal cluster is unambiguously separated from the bacterial cluster (Figure 3A), suggesting that the gene clusters in archaeal methanogens and those in the bacterial acetogens have independently evolved after separated from their common ancestral lineage. Also since there is no significant difference in the phylogenetic branching patterns of methanogenic archaea based on the sequences of the *cdh* gene clusters and the 16S rRNA genes, the methanogenic ability seems to have been primarily inherited through speciation among archaea. The tree topology also indicates that *Ca.* ‘A. autotrophicum’ diverged first from the common ancestral lineage among the nine examined bacteria. This branching pattern, though not having high bootstrap support (70%), is consistent with the results from the phylogenetic analysis of broadly conserved proteins (Figure 2A, B) although in contrast to archaeal lineage only a limited number of organisms contain these genes and the phylogenetic topology does not necessarily coincide with that of the 16S rRNA tree.

### Ancestral Pacemaker Gluconeogenic Enzyme Identified in *Ca.* ‘A. autotrophicum’

**Figure 4:**
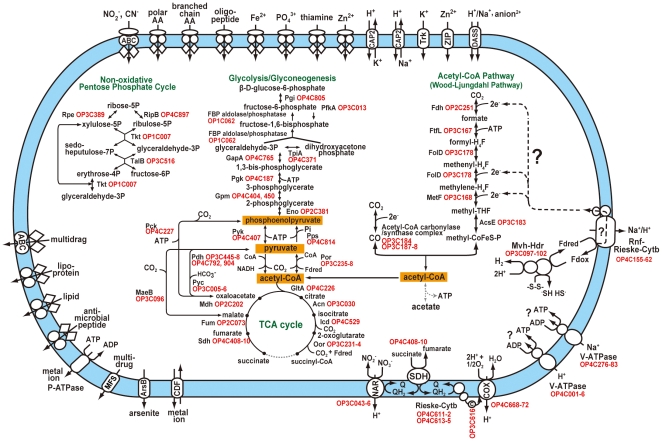
Overview of basic metabolic pathways and possible energy conservation system. Arrows indicate metabolic flows. Grey-dashed lines represent reactions for missing genes. The cytoplasmic membrane is indicated in light blue. Out of eight genes constructing V-ATPase2, two genes are likely hidden in the contig gap because OP4C001 is truncated at the end of contig. NAR, nitrate reductase; SDH, succinate dehydrogenase; Rieske, Rieske 2Fe-2S protein; Cytb, cytochrome *b*; c, cytochrome *c*; COX, cytochrome *c* oxidase; Mvh, methylviologen non-reducing hydrogenase; Hdr, heterodisulfide reductase; Rnf, electron transport complex; Fdox/Fdred, ferredoxin, oxidized and reduced form; Fdh, formate dehydrogenase; MetF, methylenetetrahydrofolate reductase; FolD, methylenetetrahydrofolate dehydrogenase; AcsE, methyltetrahydrofolate∶corrinoid/iron-sulfur protein methyltransferase; Acs, acetyl-CoA synthetase; Pgi, phosphoglucose isomerase ; Fbp, fructose-1,6-bisphosphatase I; PfkA, 6-phosphofructokinase; TpiA, triose phosphate isomerase; Gap, glyceraldehyde 3-phosphate dehydrogenase; Pgk, phosphoglycerate kinase; Gpm, phosphoglyceromutase; Eno, enolase; Pyk, pyruvate kinase; Pps, phosphoenolpyruvate synthase; Pdh, pyruvate dehydrogenase; Por, pyruvate ferredoxin oxidoreductase; GltA, citrate synthase; Acn, aconitase; Icd, isocitrate dehydrogenase; Oor, 2-oxoglutarate ferredoxin oxidoreductase; Fum, fumarase; Mdh, malate dehydrogenase; Pck, phosphoenolpyruvate carboxykinase (ATP); Pyc, pyruvate carboxylase; MaeB, malic enzyme; Ppe, ribulose phosphate 3-epimerase; RpiB, allose-6-phosphate isomerase/ribose-5-phosphate isomerase B; Tkt, transketolase; TalB, transaldolase B; Pi, phosphate; Q/QH2, quinone pool, oxidized and reduced form; ABC, ABC transporter; CAP2, monovalent cation∶proton antiporter-2 family; Trk, K^+^ transporter family; ZIP, zinc-iron permease family; DASS, divalent anion∶Na^+^ symporter family; MFS, major facilitator family; CDF, cation diffusion facilitator family; P-ATPase, P-type ATPase superfamily; ArsB, arsenite-antimonite efflux family.

Recently, it has been proposed that the heat-stable and bifunctional fructose 1,6-bisphosphate (FBP) aldolase/phosphatase highly conserved in virtually all archaeal groups as well as the deeply branching bacterial lineages represents the pacemaking ancestral gluconeogenic enzyme and that in evolution gluconeogenesis preceded glycolysis [26]. Apparently, *Ca.* ‘A. autotrophicum’ possesses all genes (OP4C187, 371, 404, 407, 450, 765, 805, 814, OP2C381, and OP3C013) related to glycolysis/gluconeogenesis, evidenced by the existence of bifunctional FBP aldolase/phosphatase (OP1C062) (Figure 4). The OP1C062 was significantly similar to those from the species within phyla such as *Deinococcus-Thermus* (62%-identity), *Thaumarchaeota* (55%), *Crenarchaeota* (54%), and *Euryarchaeota* (51%). In most Archaea that do not grow on sugars, the FBP aldolase/phosphatase is the only candidate gene for both FBP aldolase and FBP phosphatase. It has been confirmed that this bifunctionarity ensures that heat-labile triosephosphates are quickly removed and trapped in stable fructose 6-phosphate, rendering gluconeogenesis unidirectional [26].

We conducted a phylogenetic analysis using a sequence alignment of FBP aldolase/phosphatase to estimate the phylogenetic position of that found in *Ca.* ‘A. autotrophicum’. A phylogenetic tree based on the FBP aldolase/phosphatase conserved in 17 bacterial species including *Ca.* ‘A. autotrophicum’ and 28 archaeal species indicated that the FBP aldolase/phosphatase family diverges into two major clusters, cluster A containing *Euryarchaeota* and *Crenarchaeota* and cluster B containing the OP1 species and *Thermus thermophiles*, from a prokaryotic common ancestor (Figure S4). Although the phylogenetic position of *Ca.* ‘A. autotrophicum’ is near *Thermus thermophilus* within *Deinococcus-Thermus*, which is consistent with those in the broadly conserved proteins and the 16S rRNA gene, majority of bacterial and archaeal lineages are included in the cluster A and thus the topology is quite different from that of ACDS complex. In fact, the topology must be explained with a complex scenario including ancient duplications and/or horizontal transfers. At any rate, the observation does not contradict the idea that *Ca.* ‘A. autotrophicum’ has retained the ancient functionality of the gluconeogenic enzyme. Moreover, it has been experimentally confirmed that mesophilic *Cenarchaeum* enzyme in cluster B also had bifunctionarity and was heat-stable at 70°C as well as *Thermus thermophiles* [26].

### Basic Metabolism and Potential Energy Conservation Deduced from the Genome of *Ca.* ‘A. autotrophicum’

All genes (OP2C073, 202, OP3C030, 231–234, OP4C226, 408–410, 529) necessary for a complete tricarboxylic acid (TCA) cycle except for the *sucE* gene, encoding a subunit of succinyl-CoA synthetase that is necessary for the catalytic conversion between succinyl-CoA and succinate were identified in the composite genome. Thus, the TCA cycle may functions through a non-cyclic branched pathway and this incomplete TCA cycle seems to be used just for biosynthetic purposes, similarly to that in other autotrophic microbes. In the reductive direction, it is thought that acetyl-CoA is presumably converted into pyruvate and phosphoenolpyruvate by pyruvate ferredoxin oxidoreductase (OP3C235–8) and phosphoenolpyruvate synthase (OP4C814), respectively. In addition, the OP1 species was found to carry basic genes (OP1C007, OP3C389, 516, OP4C897) involved in the non-oxidative pentose phosphate pathway, which mediates the pentose production necessary for nucleic acid synthesis, as shown in Figure 4.

When *Ca.* ‘A. autotrophicum’ chemolithoautotrophically grows on H_2_/CO_2_, no net ATP is generated at the substrate level through the acetyl-CoA pathway. The process of transferring electrons from H_2_ to CO_2_ to generate a methyl group (Figure 4) involves one or more coupling sites that generate an ion gradient across the plasma membrane; however, the precise coupling sites are not yet known, even in the well-characterized acetogens [27]. *Ca.* ‘A. autotrophicum’ possesses the genes (OP3C097–102) encoding a multienzyme complex (MvhADG-HdrABC) that is composed of F420-non-reducing hydrogenase and heterodisulfide reductase. It has been shown experimentally that this multienzyme complex catalyzes the heterodisulfide-dependent reduction of ferredoxin with H_2_ by an electron bifurcation coupling mechanism in methanogenic archaea [28]. The reduced ferredoxin may be utilized for reductive reaction at the three possible sites of acetyl-CoA pathway (Figure 4). To generate proton or sodium ion gradients coupling to oxidation of the reduced ferredoxin for energy conservation in acetogenesis, a sort of pump is necessary and a membrane associated ferredoxin:NAD^+^-oxidoreductase (Rnf) complex was identified in *Ca.* ‘A. autotrophicum’ as one of good candidates. The Rnf complex has been identified in the genomes of a number of bacteria and several archaea [29] and it has been demonstrated experimentally that the Rnf complex from *Acetobacterium woodii* mediates electrogenetic Na^+^ translocation coupling to anaerobic respiration with caffeate as an electron acceptor [30]. Six genes encoding the Rnf complex (*rnfCDGEAB*) from *Ca.* ‘A. autotrophicum’ were found to form an operon-like gene cluster (OP4C155-0162) with two additional genes for membrane-bound Rieske and cytochrome *b* proteins.

The electrochemical ion potential generated across the membrane is generally utilized for ATP synthesis. *Ca.* ‘A. autotrophicum’ possesses two types of V-ATPases, H^+^ (OP4C001–006) and Na^+^ (OP4C276–283) transporting V-ATPases (Figure 4 and Figure S5) whereas F_1_F_0_-ATPase has not been identified in *Ca.* ‘A. autotrophicum’ and is also missing in *Thermus thermophiles* and *Deinococcus radiodurans* [31]. On the other hand, it has been known that the V-ATPase from *Thermus thermophilus* synthesizes ATP with proton electrochemical potential [32] whereas the activity of ATP synthesis is not experimentally confirmed in other bacteria. Thus, either or both V-ATPases may work as the alternative enzyme for ATP synthesis in *Ca.* ‘A. autotrophicum’. Actually, it has been reported that approximately 1500 ppm of hydrogen in the gas components and 500 mg/l of sodium ion was detected in the hot water near the sampling site [20].

In aerobic bacteria, O_2_^−^ and H_2_O_2_ lead to oxidative stress, but they are effectively removed by the enzymes superoxide dismutase (SOD) and catalase, respectively. Although these enzymes are often absent in anaerobic bacteria, it is known that some strict anaerobes are able to not only survive and also tolerate oxidative stress by using these enzymes [33]. In the case of *Ca.* ‘A. autotrophicum’, two SODs (OP3C460 and OP4C127) were found in the composite genome. On the other hand, a potential aerobic respiratory chain consisting of cytochrome *c* oxidase (OP4C668-72), Rieske protein, cytochrome *b* (OP4C611-12 and OP4C613-15), cytochrome *c* (OP3C616), and succinate dehydrogenase (OP4C408-10) was found in the composite genome of OP1 species (Figure 4). In addition, *Ca.* ‘A. autotrophicum’ carries genes encoding nitrate reductase (OP3C043-46) and dimethyl sulfoxide reductase (OP3C048-50) as another candidates of electron acceptor.

## Discussion

Prebiotic cellular and biochemical evolution in an alkaline hydrothermal vent chimney was proposed to involve the acetyl-CoA pathway of CO_2_ fixation [10], [11]. In addition, acetogenesis and methanogenesis enabled by this pathway have been also proposed to be the ancestral forms of energy metabolism in the first free-living bacterial and archaeal ancestors [10]. However, these hypotheses remain controversial because the acetyl-CoA pathway has not yet been found in deeply branching bacterial lineages in contrast to archaeal lineages. In this study, we found the acetyl-CoA pathway in the composite genome of one of OP1 phylotypes, a presently uncultivated bacterium within candidate division OP1. We designated this OP1 phylotype as *Candidatus* ‘Acetothermus autotrophicum’. The description of ‘A. autotrophicum‘ is as follows: ‘Acetothermus’ (A.ce.to.ther'mus. L.n. acetum vinegar; Gr. Adj. thermos hot; M.L. masc. n. Acetothermus indicates a vinegar organism living in hot places.) and ‘autotrophicum’ (au.to.tro'phi.cum. Gr. pron.autos self; Gr. adj. trophikos nursing, tending or feeding; N.L. neut. adj. autotrophicum self-nursing or self-feeding). The phylogenetic position of *Ca.* ‘A. autotrophicum’ based on the concatenated alignment of broadly conserved proteins among prokaryotic species was confirmed to branch deeply similar to the 16S rRNA tree.

Phylogenetic analysis using the concatenated alignment of five proteins comprising the ACDS complex, which is the core enzymes of the acetyl-CoA pathway, indicates that these genes in *Ca* ‘A. autotrophicum’ diverged first from the bacterial common ancestral lineage although in contrast to the archaeal lineage only very limited organisms possess the acetyl-CoA pathway in the bacterial lineage. In addition, the acetogenic phenotype has been maintained in the only limited taxon like class *Clostridia*, and just two of the species are acetogenic: *Moorella thermoacetica* [34] and C*arboxydothermus hydrogenoformans*, which is the potential acetogen possessing acetate kinase and phosphate acetyltransferase like other acetogens [35] among the species whose genome sequences have been completed. This is probably because most of the modern bacteria possessing the acetyl-CoA pathway have diversified by evolving energy generation systems more efficient than acetogenesis such as sulphate and iron reduction and dechlorination.

When acetyl-CoA generated through the acetyl-CoA pathway serves as a biosynthetic starting material, gluconeogenesis is indispensable pathway to produce various sugars and saccharides from acetyl-CoA. *Ca.* ‘A. autotrophicum’ possesses the enzyme 62%-identical to thermostable bifunctional FBP aldolase/phosphatase of *Thermus thermophilus* rendering gluconeogenesis unidirectional. As with the archaeal cases, ‘A. autotrophicum’ may use this enzyme solely under gluconeogenesis but not under glycolytic conditions because we could not identify any sugar-related transporter in the composite genome of *Ca.* ‘A. autotrophicum’. Considering that the FBP aldolase/phosphatase is highly conserved in virtually all archaeal groups and the deeply branching bacteria (although the detailed phylogenetic relationship of this family is not so clear), the FBP aldolase/phosphatase gene may have been retained in the *Ca.* ‘A. autotrophicum’ genome together with the acetyl-CoA pathway genes throughout the evolution.

The hot aquifer water stream of the Japanese epithermal mine where *Ca.* ‘A. autotrophicum’ lives, predominantly in microbial mats, represents a reductive state with the oxidation-reduction potential from −85 mV to −130 mV, but some dissolved oxygen was detected suggesting the likely presence of oxygenated ground water. Based on the reconstructed gene repertoire of *Ca.* ‘A. autotrophicum’, its primary energy and carbon metabolic pathways are predicted to be chemolithoautotrophic acetogenesis. Also, the gene repertoire indicated potential of heterotrophic O_2_- and NO_3_-respiration since autotrophic CO_2_ fixation pathways under aerobic/microaerobic conditions such as the Calvin-Benson cycle, the reductive TCA cycle and the 3-hydroxypropionate/malyl-CoA cycle [36] were not identified in the composite genome although *Ca.* ‘A. autotrophicum’ possesses a potential aerobic respiratory chain consisting of cytochrome *c* oxidase, Rieske protein, cytochrome *b*, cytochrome *c*, and succinate dehydrogenase. In agreement with these observations, *Ca*. ‘A. autotrophicum’ carries genes encoding several amino acid ABC transporters necessary for the uptake of amino acids as carbon sources in the heterotrophy (Figure 4).

We can also deduce to some extent the role of *Ca.* ‘A. autotrophicum’ in the metabolism of the microbial mat community from the rest of our metagenomic data. Among the phylotypes identified in the metagenomic library of the microbial mat community (Figure S6), the phylotypes closely related to *Methylohalobius crimeensis*, *Hydrogenobacter thermophilus*, *Hydrogenophilus thermoluteolus*, hot water crenarchaeotic group (HWCG) I and III likely use CH_4_ and CO_2_ supplied from the geothermal aquifer in chemolithoautotrophic and methanotrophic metabolic pathways; although the abundance of clones from *H. thermophilus*, *H. thermoluteolus*, and HWCGIII is very low. However, these results differ from those of the previous study based on the analysis of bacterial 16S rRNA genes in which the OP1 phylotype was minor in the microbial mat community [20]. In addition, it has also been reported in previous studies that the microbial mat community is dominated by the potentially chemolithoautotrophic HWCGIII (*Ca.* ‘Nitrosocaldus’ sp.) and HWCGI (*Ca.* ‘Caldiarchaem subterraneum’) and thermophilic methanotrophs [21], [22], [37]. Considering the numerical abundance of *Ca.* ‘A. autotrophicum’ found in this study, we speculate that anaerobic acetogenesis may be an opportunistic option for *Ca.* ‘A. autotrophicum’ when its microhabitat in the oxic-anoxic interface becomes anaerobic organics-depleted or aerobic-enriched. Accordingly, chemolithoautotrophic acetogenesis in *Ca.* ‘A. autotrophicum’ may serve as the primary energy and carbon metabolic pathway and contribute to the primary production of the whole microbial community in the anaerobic organics-depleted state.

These findings do not necessarily mean that *Ca.* ‘A. autotrophicum’ conducting acetogenesis through the ancestral acetyl-CoA pathway is a living fossil because although some of genes early diverged from a universal last common ancestor remain in the genome, *Ca.* ‘A. autotrophicum’ could be conceivably evolved independently in the fluctuating thermal environments between anaerobic organics-depleted and aerobic-enriched states with keeping a part of primordial genes for basic energy and carbon metabolisms in the limited microhabitat similar to the ancient hydrothermal environments. Our metagenomic approach will be helpful to gather the genomic information of further uncultivated deeply branching bacteria, which may harbour in the limited hydrothermal environments and also provide important clues to make the deeply branching bacteria cultivable.

## Materials and Methods

### DNA Sequencing and Assembly

The paired-end sequences of 5,280 previously prepared [21] fosmid clones were sequenced using an ABI PRISM 3730 DNA sequencer (Applied Biosystems); 5,964 readable sequences from 3,375 clones were used in this study. Among the 3,375 fosmid clones, 136 randomly selected clones and 15 clones carrying the 16S rRNA gene confirmed by dot blot hybridisation were sequenced by the Sanger method using ABI PRISM 3730 and MegaBase1000 (GE Healthcare, Japan) DNA sequencers. The Sanger sequence reads were assembled using Phrap with default parameters. The final gaps in each clone were closed by direct sequencing of the PCR products. The nucleotide sequences of 176 clones selected as genomic fragments derived from the OP1 species (*Ca.* ‘Acetothermus autotrophicum’) based on codon usage were determined by pyrosequencing using a Genome Sequencer FLX System (Roche Diagnostics) according to the manufacturer's protocol. The pyrosequencing reads were assembled using a 454 Newbler assembler version 1.1.02.15 at default settings; the constructed contigs were assembled again with 34 fosmid clones from *Ca.* ‘A. autotrophicum’ sequenced by the Sanger method to reconstruct the genome. All sequences determined in this study have been deposited in DDBJ/EMBL/GenBank with the following accession numbers: AP011626 to AP011799 and AP011903 for the sequences of the completely determined fosmid clones, AG993735 to AG999698 for the end sequences of 3,375 fosmid clones, AP011800 to AP011803 for the sequences of the *Ca.* ‘A. autotrophicum’ contigs, and DRP000009 for the sequences and quality scores from the pyrosequencing reads. All genetic information from *Ca.* ‘A. autotrophicum’ and other fosmid clones sequenced in this study are also publically accessible in our “ExtremoBase” on the JAMSTEC database web site (http://www.jamstec.go.jp/gbrowser/cgi-bin/top.cgi).

### Genome Annotation and Pathway Analysis

CDSs were predicted using a combination of the Genome Gambler™ and MetaGeneAnnotator programs [38], [39]. Genes encoding tRNAs were identified by tRNA scan-SE [40], whereas rRNA genes were identified using the BLASTN program. The remaining parts of the genome were further screened to find missed CDSs by a BLASTX homology search against the NCBI nonredundant protein database and the KEGG GENES database (http://www.genome.jp/kegg/genes.html). The amino acid sequences of predicted CDSs were subjected to a BLASTP homology search against the protein databases for functional assignment. Significant homology was defined as at least 30% identity over 60% of the CDS; however, those CDSs showing <30% identity over >60% of the protein were also included, as previously described [41]. The KEGG PATHWAY [42] and MetaCyc [43] databases were used for pathway reconstruction.

### 16S rRNA Gene Analysis

The 16S rRNA genes of *Geobacillus kaustophilus* HTA 426 (AB002645) [44], hot water crenarchaeotic group (HWCGI) *Ca*, ‘Caldiarchaeum subterraneum’ (AB201309) and HWCGIII ‘Nitrosocaldus sp.’ (AB201308) [21] were used as DNA probes for dot blot hybridisation to detect bacterial and archaeal 16S rRNA genes in the fosmid clones. All fosmid DNAs were extracted from overnight cultures using a PI-200 automatic DNA extraction system (Kurabo Industries, Osaka, Japan). Dot blot hybridisation was performed with a digoxigenin-labelled DNA probe and an antidigoxigenin antibody coupled to alkaline phosphatase using a DNA labelling and detection kit (Roche Diagnostics). The 16S rRNA genes detected in the fosmid clones were amplified by PCR using a standard bacterial consensus primer set (27F-1492R; 5′-agagtttgatcctggctcag-3′, 5′-ggttaccttgttacgactt-3′) [45] and a standard archaeal consensus primer set (21F-958R; 5′-ttccggttgatccygccgga-3′, 5′-yccggcgttgamtccaatt-3′) [46]. The PCR products were sequenced by the Sanger method. The representative sequences of the 16S rRNA genes in the fosmid clones were subjected to similarity searches against the NCBI nonredundant nucleotide database (http://www.ncbi.nlm.nih.gov/) and the Ribosomal Database Project (RDP) database (http://rdp.cme.msu.edu/) using the BLASTN program. The nucleotide sequences were automatically aligned with the closely related 16S rRNA gene sequence; a phylogenetic tree was constructed by the neighbour-joining method [47] using Clustal X ver.1.83.1 [48] or by the maximum likelihood method using PHYML [49]. Bootstrap analysis was performed for 1,000 replicates to assign a confidence level to the tree topology.

### Clustering Analysis Based on Codon Usage Patterns

First, the completely sequenced fosmid clones were classified into corresponding species groups according to the codon usages of the annotated CDSs. The start and stop codons were excluded from the calculation of codon usage, and a 61-dimensional vector (a set of 61 codon frequencies) for each clone was obtained from the internal codons of all CDSs on the clone. The Euclidean distance between the codon usages was used as the distance measure, and the group average method, a simple hierarchical clustering method also known as UPGMA (Unweighted Pair Group Method with Arithmetic mean), was applied for species classification.

Because the clone-end sequences are as short as about 600 base pairs (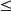
200 codons), the codon frequencies calculated from each clone-end sequence deviate markedly from the original codon frequencies observed in the entire genomic sequence. In this study, the probability that a clone-end sequence derives from a given species was calculated and used as a classifier. The number of codons appearing in the clone-end sequences follows a multinomial distribution, and the probability of obtaining the clone-end sequence 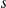 from a species 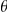 is:
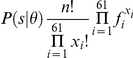
where 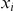 is the number of codons 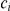 (*i* = 1,…,61) in clone-end sequence 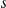, and the total number of codons is 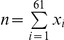. 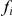 is the frequency of codon 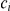 in species 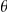
_,_ and 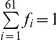. The species groups defined by the complete fosmid sequences were used as the candidate origins of the clone-end sequences, and each clone-end sequence (or paired-end sequences if available) was attributed to most probable species group.

As a preliminary clustering analysis, we tested the Euclidean distances between the codon usages of artificial fosmid sequences containing 8,000 codons made from 456 complete genomic sequences of the same or different species and then set the threshold for the same species group at 0.04 (Figure S2A). In addition, to evaluate the accuracy of the above classification method, we classified the artificial genomic fragments consisting of 100, 200 or 400 codons made from known genome sequences (Figure S1B). As a result, 89% of the fragments were correctly classified, even in the cases of 100 codons for which the Euclidean distance between the species was longer than 0.04.

### Phylogenetic Analysis of *Ca.* ‘A. autotrophicum’ from the Phylogenomic Point of View

To determine the phylogenetic position of *Ca.* ‘A. autotrophicum’ from a phylogenomic point of view, genome-wide phylogenetic analyses were performed using a concatenated alignment of protein-coding genes [50]. According to a previous study [51], 31 protein-coding genes were shared among 357 bacterial species (Table S3), of which only 16 were found in *Ca.* ‘A. autotrophicum’, suggesting that the remaining 15 genes encoding ribosomal proteins are surely hidden in just one missing fosmid clone. Sixteen proteins broadly conserved in 358 bacterial genomes, including *Ca.* ‘A. autotrophicum’, and four proteins conserved in 367 prokaryotes, including 359 bacterial and nine archaeal genomes, were used in this study. These concatenated protein sequences were aligned with the reference alignment included in the software package AMPHORA [23]. A maximum likelihood tree was constructed by RAxML [52] using the resulting concatenated alignment.

### Phylogenetic Analysis of the ACDS Complex

To clarify the phylogenetic relationship of *Ca.* ‘A. autotrophicum’ among the prokaryotic species possessing the acetyl-CoA pathway, a phylogenetic analysis was performed based on the concatenated alignment of protein sequences deduced from five genes (*acs*/*cdhA-D* and *acs*/*cdhF*) encoding the ACDS complex, the core proteins in this pathway. Orthologous gene sets in the *acs/cdh* core gene cluster were retrieved using MBGD [53]. When multiple inparalogous genes were included in an orthologous group, only the one conserved in the operon-like cluster was used for constructing the phylogram. A phylogenetic tree based on the 16S rRNA genes from the species possessing the orthologous core gene set was also constructed to compare the phylogenetic relationships based on the ACDS complex and on the 16S rRNA gene. Both maximum likelihood trees were constructed using PHYML [49].

### Ortholog Analysis

Orthologous relationships between each *Ca.* ‘A. autotrophicum’ gene and the genes of other organisms were identified by the DomClust program [54] based on input all-against-all similarities between *Ca.* ‘A. autotrophicum’ and 341 representative organisms (one genome was selected from each genus) from the MBGD database [53]. The resulting ortholog table was visualized and analyzed through the RECOG system (Uchiyama et al, unpublished).

## Supporting Information

Figure S1 **Verification of accuracy for the clustering analysis based on the codon usage patterns.**
(TIF)

Figure S2 **Correlation between the maximum growth temperature and the G+C contents of their 16S rRNA genes.**
(TIF)

Figure S3 **Biochemical reactions in acetogenesis and methanogenesis, and the organization of the gene cluster** [**55**] **.**
(TIF)

Figure S4 **Maximum-likelihood phylogenetic trees based on the sequences of FBP aldolase/phosphatase.**
(TIF)

Figure S5 **Alignment of amino acid sequences of transmembrane segment in the two types of V-type ATPases.** A: Na^+^-transporting V-ATPase [56], B: H^+^-transporting V-ATPase [57].
(TIF)

Figure S6 **Phylogenetic tree based on 16S rRNA genes found in the fosmid library.**
(TIF)

Table S1 **Orthologous gene list shared in 95% of bacteria whose genome size are more than 1.5 Mb.**
(PDF)

Table S2 **List of the prokaryotic species used for phylograms in** **Fig. 2** **.**
(PDF)

Table S3 **List of species or clone name used for phylogenetic analysis based on 16S rRNA genes.**
(PDF)
