# Supplementary figures and images for: A Deeply Branching Thermophilic Bacterium with an Ancient Acetyl-CoA Pathway Dominates a Subsurface Ecosystem
Source: PLoS One. 2012 Jan 27;7(1):e30559. doi: 10.1371/journal.pone.0030559 (PMC3267732; doi:10.1371/journal.pone.0030559)

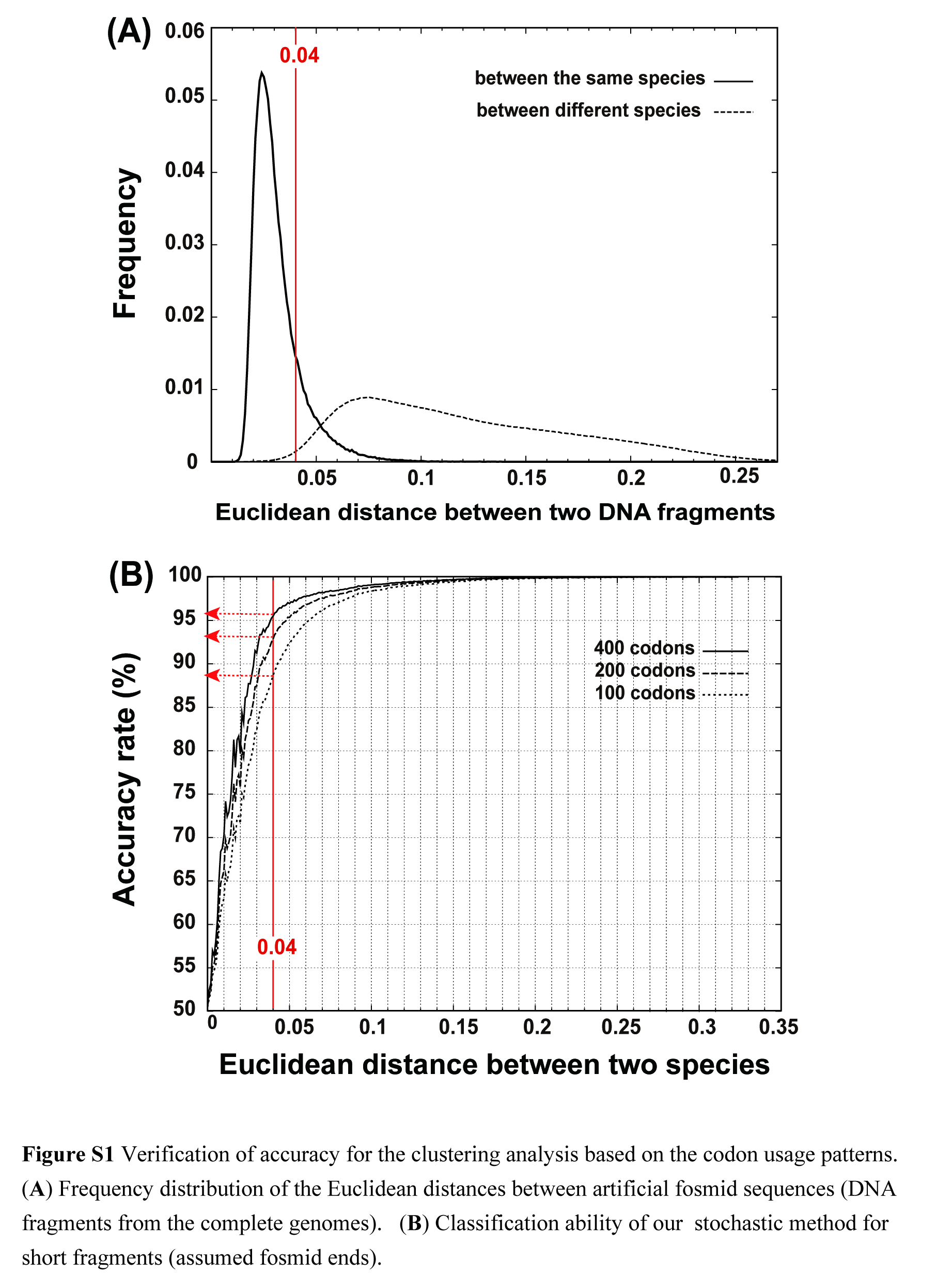

Supplement: Figure S1 — Verification of accuracy for the clustering analysis based on the codon usage patterns. (TIF) [file pone.0030559.s001.tif]

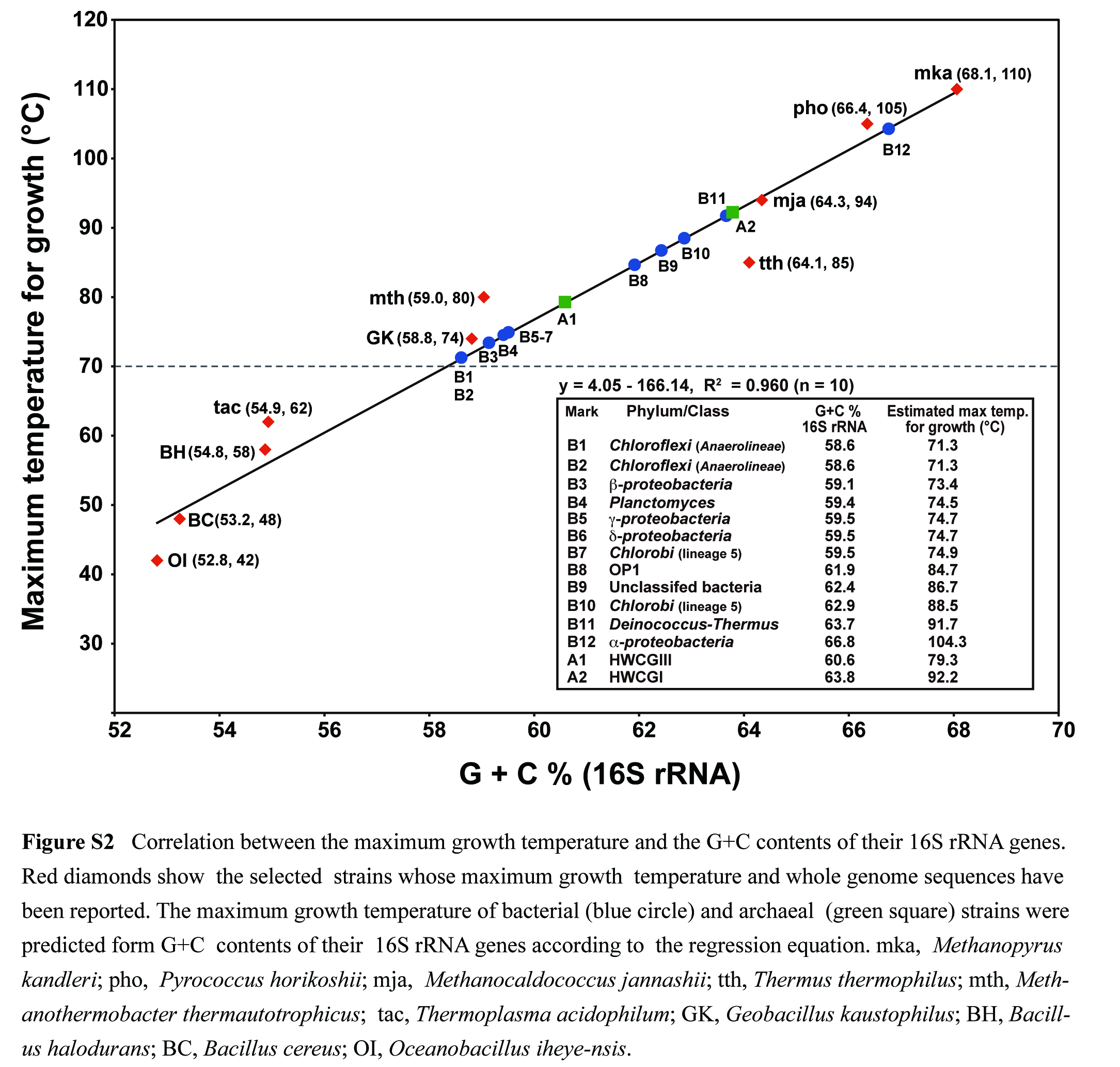

Supplement: Figure S2 — Correlation between the maximum growth temperature and the G+C contents of their 16S rRNA genes. (TIF) [file pone.0030559.s002.tif]

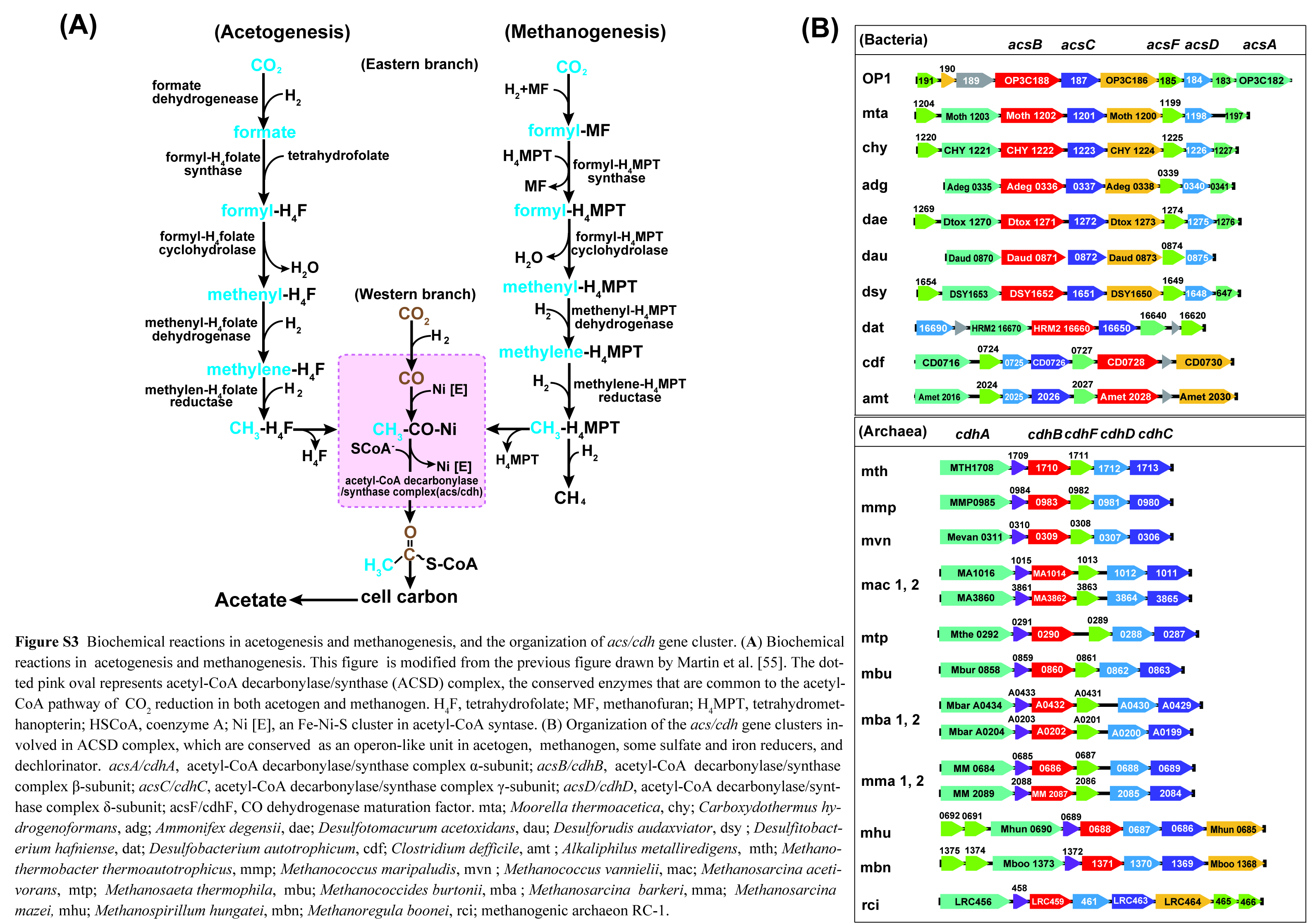

Supplement: Figure S3 — Biochemical reactions in acetogenesis and methanogenesis, and the organization of the gene cluster [55] . (TIF) [file pone.0030559.s003.tif]

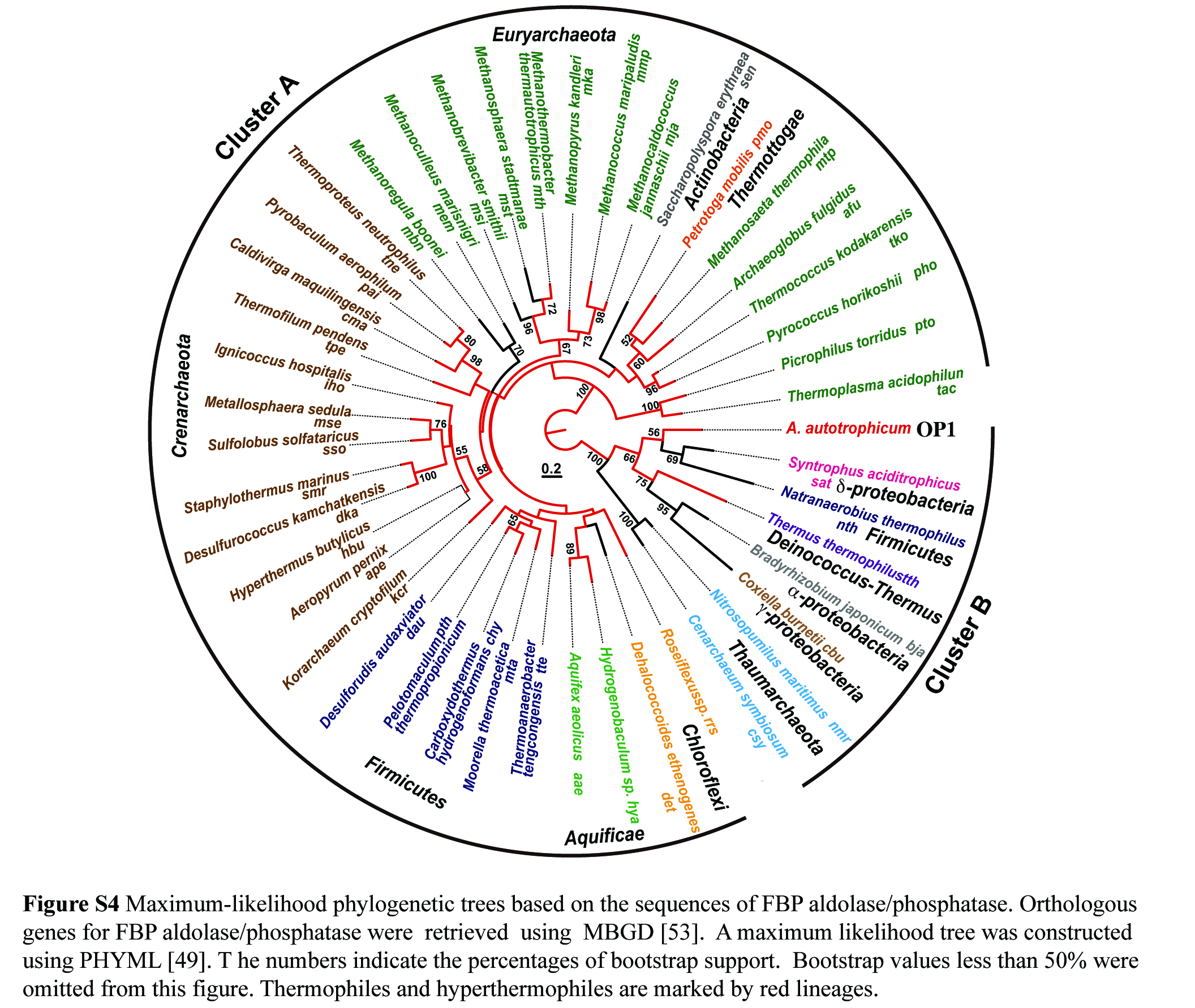

Supplement: Figure S4 — Maximum-likelihood phylogenetic trees based on the sequences of FBP aldolase/phosphatase. (TIF) [file pone.0030559.s004.tif]

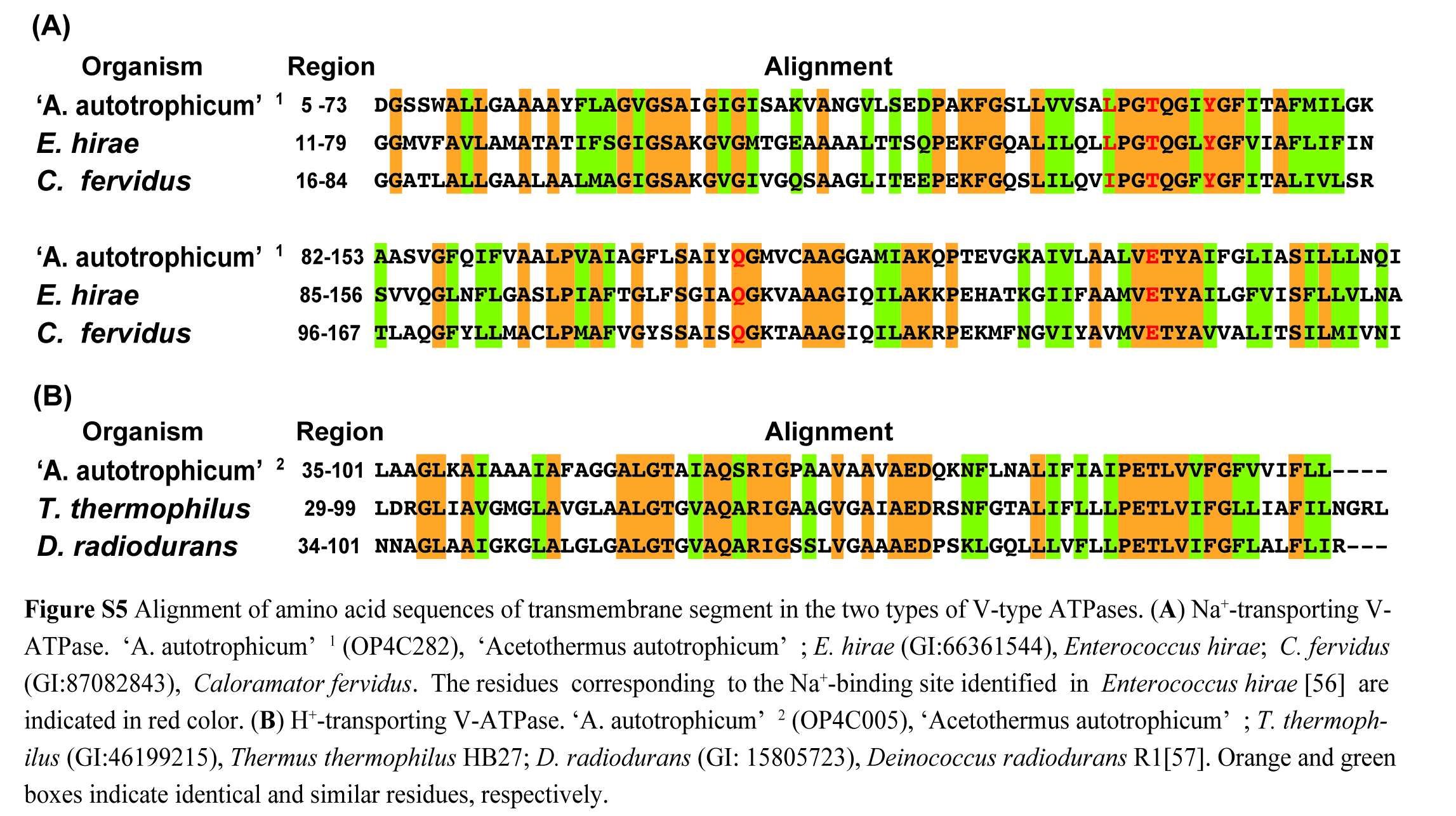

Supplement: Figure S5 — Alignment of amino acid sequences of transmembrane segment in the two types of V-type ATPases. A: Na+-transporting V-ATPase [56], B: H+-transporting V-ATPase [57]. (TIF) [file pone.0030559.s005.tif]

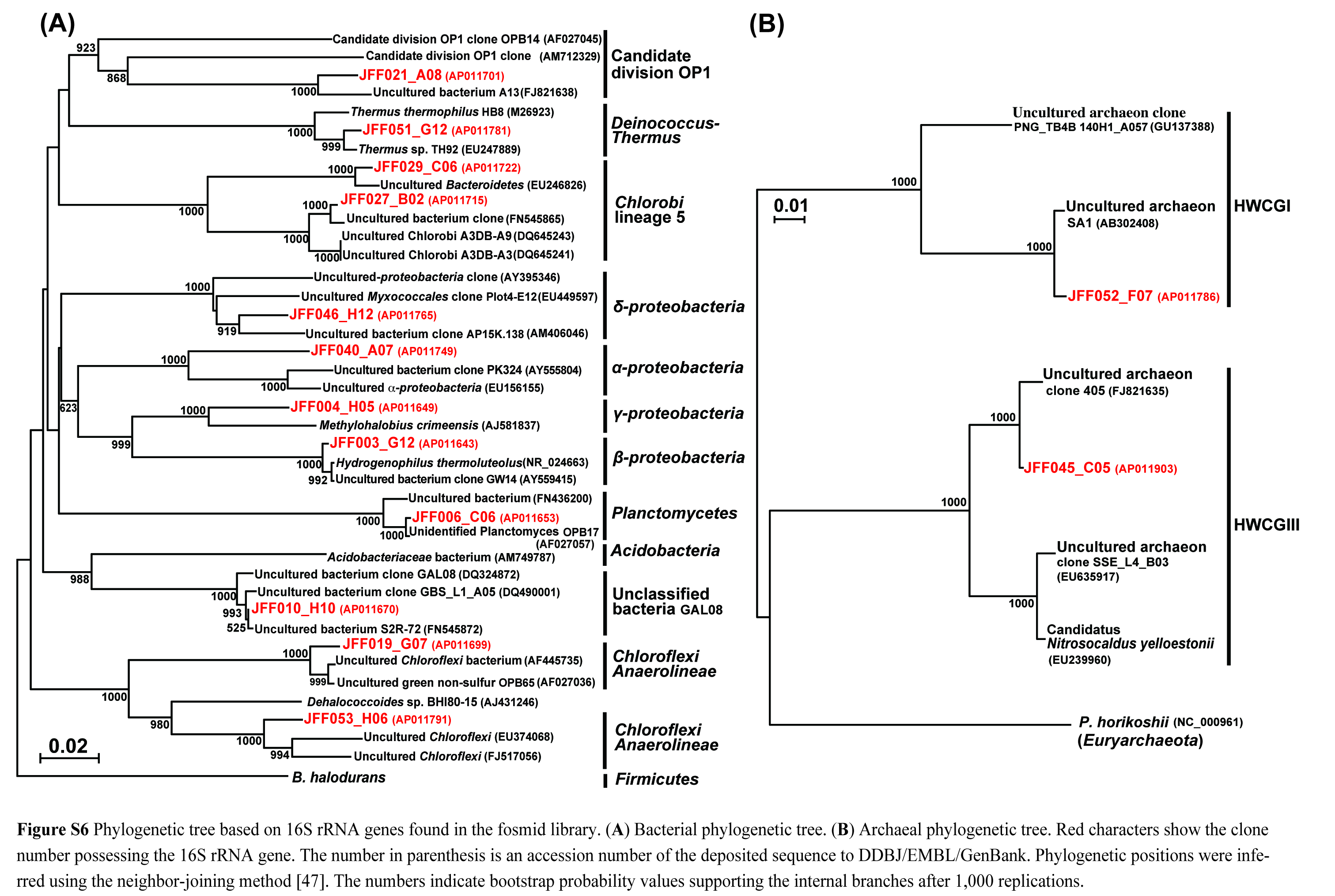

Supplement: Figure S6 — Phylogenetic tree based on 16S rRNA genes found in the fosmid library. (TIF) [file pone.0030559.s006.tif]
