# Supplementary material for: A Deeply Branching Thermophilic Bacterium with an Ancient Acetyl-CoA Pathway Dominates a Subsurface Ecosystem
Source: PLoS One. 2012 Jan 27;7(1):e30559. doi: 10.1371/journal.pone.0030559 (PMC3267732; doi:10.1371/journal.pone.0030559)
Supplement: Table S1 — Orthologous gene list shared in 95% of bacteria whose genome size are more than 1.5 Mb. (PDF) [file pone.0030559.s007.pdf]

Table S1 Orthologous gene list shared in 95%of bacteria whose genome size are more than 1.5 Mb

Ortholog ID	Cluter ID	Number found in the composite genome	Gene product
1028	C_001	0	50S ribosomal protein L18
1047	C_001	0	50S ribosomal protein L24
1070	C_001	0	DNA-directed RNA polymerase subunit alpha
575	C_001	0	50S ribosomal protein L3
592	C_001	0	30S ribosomal protein S10
599	C_001	0	50S ribosomal protein L2
603	C_001	0	30S ribosomal protein S17
610	C_001	0	30S ribosomal protein S13
615	C_001	0	30S ribosomal protein S19
617	C_001	0	50S ribosomal protein L5
622	C_001	0	30S ribosomal protein S8
628	C_001	0	50S ribosomal protein L6
634	C_001	0	50S ribosomal protein L14
636	C_001	0	50S ribosomal protein L22
649	C_001	0	50S ribosomal protein L23
659	C_001	0	Preprotein translocase subunit (SecY)
665	C_001	0	30S ribosomal protein S5
677	C_001	0	30S ribosomal protein S11
706	C_001	0	30S ribosomal protein S3
709	C_001	0	50S ribosomal protein L29
942	C_001	0	50S ribosomal protein L17
963	C_001	0	50S ribosomal protein L4
966	C_001	0	50S ribosomal protein L16
973	C_001	0	50S ribosomal protein L15
215	C_002	1	Cysteine synthase A
244	C_002	1	Cysteine synthase A
156	C_003	3	Anthranilate synthase component II
780	C_003	1	GMP synthase
4630	C_004	1	Chain elongation factor-G
609	C_004	0	30S ribosomal protein S12
637	C_004	0	30S ribosomal protein S7
1105	C_005	1	DNA-directed RNA polymerase subunit beta
507	C_005	1	DNA-directed RNA polymerase subunit beta
508	C_005	1	DNA-directed RNA polymerase subunit beta
161	C_006	0	Chaperonin GroEL protein
574	C_006	0	Co-chaperonin GroES protein
1056	C_007	0	F0F1 ATP synthase subunit delta
210	C_007	1	F0F1 ATP synthase subunit beta
815	C_007	0	F0F1 ATP synthase subunit epsilon
852	C_007	0	F0F1 ATP synthase subunit gamma
95	C_007	2	F0F1 ATP synthase subunit beta
1011	C_008	0	Preprotein translocase subunit (SecF)
995	C_008	0	Preprotein translocase subunit (SecD)
812	C_009	0	F0F1 ATP synthase subunit C
879	C_009	0	F0F1 ATP synthase subunit A
1055	C_010	1	50S ribosomal protein L21
980	C_010	1	50S ribosomal protein L27
10	C_011	5	3-carrier reductase / Short-chain dehydrogenase/reductase (SDR)
19	C_011	3	3-carrier reductase / Short-chain dehydrogenase/reductase (SDR)
41	C_011	3	3-oxoacyl-(acyl-carrier protein) reductase / Short-chain dehydrogenase/reductase (SDR)
35	C_012	5	Two-component response transcriptional regulator
4	C_012	6	Two-component response transcriptional regulator

1095	C_013	1	Leucyl-tRNA synthetase
510	C_013	1	Leucyl-tRNA synthetase
24	C_014	2	ABC transporter ATP-binding protein
25	C_014	2	ABC transporter ATP-binding protein
728	C_014	2	ABC transporter ATP-binding protein
1	C_015	14	ABC transporter, ATP-binding protein-related protein
2	C_015	12	ABC transporter, ATP binding protein
3	C_015	7	ABC transporter, ATP binding protein
6892	C_015	40	ABC transporter, ATP binding protein related protein
1126	C_016	1	Transcription antitermination protein (NusG)
590	C_016	1	50S ribosomal protein L1
705	C_016	1	50S ribosomal protein L11
883	C_016	1	50S ribosomal protein L7/L12
984	C_016	1	50S ribosomal protein L10
354	C_017	1	Alanyl-tRNA synthetase
693	C_017	1	Alanyl-tRNA synthetase
1041	C_018	1	50S ribosomal protein L20
1144	C_018	1	50S ribosomal protein L35
1114	C_019	1	Polynucleotide phosphorylase/polyadenylase
139	C_019	1	30S ribosomal protein S1
140	C_019	2	Polynucleotide phosphorylase/polyadenylase
1111	C_020	1	30S ribosomal protein S6
906	C_020	1	30S ribosomal protein S18
136	C_021	2	Ribosomal large subunit pseudouridine synthase
235	C_021	1	Ribosomal large subunit pseudouridine synthase
1348	C_022	1	Translation initiation factor IF-2
804	C_022	1	Transcription elongation factor (NusA)
1051	C_023	0	Elongation factor Ts
589	C_023	1	30S ribosomal protein S2
561	C_024	1	30S ribosomal protein S9
569	C_024	0	50S ribosomal protein L13
1062	C_025	1	DNA polymerase I
686	C_025	1	DNA polymerase I
54	S_0054	0	Type II secretion system protein E
70	S_0070	2	Aspartate aminotransferase
74	S_0074	1	Cation diffusion facilitator family transporter
77	S_0077	3	Glucose-1 phosphate transferase
79	S_0079	2	Heavy metal translocating P-type ATPase
88	S_0088	0	HAD hydrolase family protein
90	S_0090	2	Dihydrolipoamide dehydrogenase
98	S_0098	2	Two-component response regulator transcriptional regulator protein
99	S_0099	3	GTP-dependent nucleic acid-binding protein (EngD)
103	S_0103	1	Era, Era/TrmE GTP-binding protein
105	S_0105	1	Thioredoxin protein
107	S_0107	1	MscS Mechanosensitive ion channel protein
115	S_0115	2	TRNA/rRNA methyltransferase
121	S_0121	1	Metallo-beta-lactamase domain-containing protein
137	S_0137	1	Phosphoglucosamine mutase mutase / phosphomannomutase
144	S_0144	1	Cysteine desulfurase, aminotransferase, class V
147	S_0147	5	Integration host factor, DNA-binding protein, alpha subunit
148	S_0148	1	Hemolysin protein containing CBS domain
176	S_0176	1	GTP-binding protein (LepA)
177	S_0177	1	Cell division protein FtsH, ATP-dependent
180	S_0180	1	1-acyl-sn-glycerol-3-phosphate acyltransferase
183	S_0183	2	DNA polymerase III subunits gamma and tau
201	S_0201	0	Dihydrodipicolinate synthase

216	S_0216	1	MiaB-like tRNA modifying enzyme
220	S_0220	1	Ferric uptake regulation protein ferric uptake regulator
226	S_0226	1	Elongation factor Tu
242	S_0242	1	Branched-chain amino acid aminotransferase
247	S_0247	2	UDP-N-acetylmuramoylalanyl-D-glutamate--2, 6-diaminopimelate ligase/UDP-N-tripeptide:D-alanyl-D ligase
250	S_0250	2	Peptide chain release factor 1
254	S_0254	1	Glyceraldehyde-3-phosphate dehydrogenase
260	S_0260	3	Phospho-N-acetylmuramoyl-pentapeptide- transferase
262	S_0262	1	RodA, rod cell shape determining protein
265	S_0265	0	Protein tRNA dihydrouridine synthase Tim barrel
266	S_0266	0	Histidinol-phosphate aminotransferase
272	S_0272	1	ATP-dependent DNA helicase, UvrD/REP
290	S_0290	2	3-oxoacyl-(acyl carrier protein) synthase III
294	S_0294	1	Glucosamine-1-phosphate N-acetyltransferase / UDP-N-acetylglucosamine pyrophosphorylase
297	S_0297	1	DNA gyrase / DNA topoisomerase subunit A
306	S_0306	1	3-oxoacyl-(acyl carrier protein) synthase II
319	S_0319	0	Methionine aminopeptidase, type I
329	S_0329	1	N5-glutamine S-adenosyl-L-methionine-dependent methyltransferase
340	S_0340	0	Phosphoribosylglycinamide formyltransferase
342	S_0342	0	DNA gyrase / DNA topoisomerase subunit B
350	S_0350	3	RNA polymerase sigma factor (RpoD)
364	S_0364	0	Aspartate kinase
367	S_0367	1	Histidyl-tRNA synthetase
371	S_0371	0	Oxygen-independent coproporphyrinogen III oxidase
375	S_0375	1	DNA repair protein RadA
382	S_0382	1	Transketolase
383	S_0383	1	Sua5/YciO/YrdC/YwlC family translation factor
389	S_0389	1	TatD-related deoxyribonuclease family protein
397	S_0397	1	Ribose-phosphate pyrophosphokinase
399	S_0399	1	Ribonucleotide-diphosphate reductase subunit alpha
400	S_0400	1	DNA polymerase III subunit alpha
411	S_0411	1	DO serine protease
418	S_0418	2	ATP-dependent Clp protease, ATP-binding subunit (ClpB)
423	S_0423	2	Recombinase A
429	S_0429	0	Inosine-5'-monophosphate dehydrogenase
438	S_0438	1	Peptide deformylase
440	S_0440	1	Shikimate 5-dehydrogenase
442	S_0442	1	Single-strand DNA-binding protein
450	S_0450	1	TRNA nucleotidyltransferase/poly(A) polymerase protein
452	S_0452	1	Glucosamine--fructose-6-phosphate aminotransferase
455	S_0455	1	Serine hydroxymethyltransferase
456	S_0456	0	Adenylate kinase
463	S_0463	0	ATP-dependent Clp protease proteolytic subunit
469	S_0469	1	Dihydroorotate dehydrogenase 2
473	S_0473	1	Acyl carrier protein
482	S_0482	0	Undecaprenyl pyrophosphate synthetase synthase
486	S_0486	4	Signal peptidase I
492	S_0492	1	Adenylosuccinate lyase
498	S_0498	2	CDP-diacylglycerol--glycerol-3-phosphate 3-phosphatidyltransferase
501	S_0501	1	Tryptophanyl-tRNA synthetase
511	S_0511	0	30S ribosomal protein S4
512	S_0512	1	UDP-N-acetylmuramate--L-alanine ligase
514	S_0514	2	Putative ATP-binding protein involved in chromosome partitioning
531	S_0531	1	Inorganic polyphosphate/ATP-NAD kinase
534	S_0534	1	Phenylalanyl-tRNA synthetase, alpha subunit

535	S_0535	1	Tyrosyl-tRNA synthetase
540	S_0540	1	Phosphopyruvate hydratase
541	S_0541	0	F0F1 ATP synthase subunit B
542	S_0542	1	Triosephosphate isomerase
547	S_0547	1	Cell division protein (FtsZ)
556	S_0556	1	Glutamyl-tRNA synthetase
558	S_0558	1	Molecular chaperone protein (DnaK)
563	S_0563	0	TRNA pseudouridine synthase A
566	S_0566	1	Cysteinyl-tRNA synthetase
571	S_0571	2	Prolyl-tRNA synthetase
607	S_0607	1	Methionyl-tRNA synthetase
619	S_0619	1	Isoleucyl-tRNA synthetase
623	S_0623	0	Aspartate semialdehyde dehydrogenase
624	S_0624	1	Dimethyladenosine transferase
625	S_0625	1	Histidine triad family protein / HIT family protein
635	S_0635	1	NAD synthetase
639	S_0639	1	Arginyl-tRNA synthetase
642	S_0642	1	Seryl-tRNA synthetase
644	S_0644	1	Putative DNA-binding/iron metalloprotein/AP endonuclease
661	S_0661	1	D-alanine--D-alanine ligase
664	S_0664	1	Bifunctional protein: methylenetetrahydrofolate dehydrogenase; methenyltetrahydrofolate cyclohydrolase
666	S_0666	1	Phosphoglycerate kinase
672	S_0672	1	Chaperone protein (DnaJ)
676	S_0676	1	Thymidylate kinase
678	S_0678	0	(p)ppGpp synthetase I guanosine-3',5' 3'-pyrophosphohydrolase
688	S_0688	1	Valyl-tRNA synthetase
692	S_0692	1	Replicative DNA helicase
694	S_0694	1	Methionyl-tRNA formyltransferase
697	S_0697	1	Carbamoyl phosphate synthase large subunit
698	S_0698	1	Shikimate kinase
707	S_0707	1	CTP synthetase
712	S_0712	1	Phenylalanyl-tRNA synthetase subunit beta
715	S_0715	1	Biotin--acetyl-CoA-carboxylase ligase protein
716	S_0716	1	Heat shock protein (GrpE)
731	S_0731	1	Orotate phosphoribosyltransferase
733	S_0733	1	Signal recognition particle protein
739	S_0739	0	TRNA pseudouridine synthase B
745	S_0745	1	Ribonuclease HII
746	S_0746	1	S-adenosylmethionine synthetase
747	S_0747	1	Phosphatidate cytidylyltransferase
749	S_0749	1	Ribulose-phosphate 3-epimerase
751	S_0751	1	Carbamoyl phosphate synthase small subunit
752	S_0752	1	Adenylosuccinate synthetase
756	S_0756	1	Phosphopantothenoylcysteine decarboxylase/phosphopantothenate--cysteine ligase
757	S_0757	1	Non-canonical purine NTP pyrophosphatase, rdgB/HAM1 family protein
761	S_0761	1	Guanylate kinase
762	S_0762	1	Malonyl CoA-acyl carrier protein transacylase
768	S_0768	2	FolC bifunctional protein: folylpolyglutamate synthase dihydrofolate
772	S_0772	1	Elongation factor P
776	S_0776	0	Amidophosphoribosyltransferase
779	S_0779	1	Aspartate carbamoyltransferase catalytic subunit
784	S_0784	0	Phosphoribosylaminoimidazole-succinocarboxamide synthase
790	S_0790	1	Putative inner membrane protein translocase component (YidC)
803	S_0803	1	Peptidase M50, putative membrane-associated zinc
807	S_0807	1	UDP-N-acetylglucosamine 1-carboxyvinyltransferase

808	S_0808	2	Alanine racemase
811	S_0811	1	3-phosphoshikimate 1-carboxyvinyltransferase
822	S_0822	1	Phosphoribosylamine--glycine ligase
828	S_0828	1	50S ribosomal protein L33
836	S_0836	1	Lipoprotein signal peptidase
841	S_0841	1	Pyrroline-5-carboxylate reductase
851	S_0851	0	Uridylate kinase
853	S_0853	1	Phosphoribosylaminoimidazole carboxylase, catalytic subunit
857	S_0857	1	Thioredoxin reductase
868	S_0868	0	Phosphoribosylaminoimidazole synthetase
870	S_0870	1	NAD(P)H-dependent glycerol-3-phosphate dehydrogenase
873	S_0873	1	Signal recognition particle-docking protein FtsY
877	S_0877	0	Translation initiation factor IF-1
884	S_0884	0	30S ribosomal protein S14
885	S_0885	0	Phosphoribosylformylglycinamidine synthase I
887	S_0887	0	Phosphoribosylformylglycinamidine synthase II
889	S_0889	1	Excinuclease ABC subunit A
890	S_0890	1	Cytidylate kinase
896	S_0896	1	Ribonuclease III
904	S_0904	1	Chorismate synthase
907	S_0907	1	TRNA delta(2)-isopentenylpyrophosphate transferase
909	S_0909	0	Glucose-6-phosphate isomerase
920	S_0920	0	Protein of unknown function DUF28
932	S_0932	1	50S ribosomal protein L31
933	S_0933	1	50S ribosomal protein L28
938	S_0938	0	ATP-dependent protease ATP-binding subunit (ClpX)
940	S_0940	1	Preprotein translocase subunit SecA
941	S_0941	1	Protein RibF: riboflavin kinase; FMN adenylyltransferase
944	S_0944	1	Prolipoprotein diacylglyceryl transferase
949	S_0949	1	Dephospho-CoA kinase
957	S_0957	1	Bifunctional phosphoribosylaminoimidazolecarboxamide formyltransferase/IMP cyclohydrolase
971	S_0971	0	DNA topoisomerase I
974	S_0974	1	50S ribosomal protein L19
975	S_0975	1	Peptidyl-tRNA hydrolase
976	S_0976	1	2-amino-4-hydroxy-6- hydroxymethyldihydropteridine pyrophosphokinase
977	S_0977	1	TRNA (5-methylaminomethyl-2-thiouridylate)-methyltransferase
981	S_0981	1	30S ribosomal protein S15
986	S_0986	1	DNA polymerase III, beta subunit
993	S_0993	1	Ribosome recycling factor
996	S_0996	1	Alanine racemase domain-containing protein
1001	S_1001	1	Peptidoglycan glycosyltransferase/penicillin-binding protein
1003	S_1003	1	30S ribosomal protein S16
1010	S_1010	1	Aspartyl-tRNA synthetase
1018	S_1018	1	Excinuclease ABC subunit B
1020	S_1020	2	DprA/SMF protein DNA processing
1026	S_1026	1	Excinuclease ABC subunit C
1031	S_1031	0	16S ribosomal RNA methyltransferase (RsmE)
1032	S_1032	0	Dihydrodipicolinate reductase
1043	S_1043	1	S-adenosyl-methyltransferase (MraW)
1045	S_1045	0	Diaminopimelate decarboxylase
1049	S_1049	1	Translation initiation factor IF-3
1059	S_1059	1	TRNA(Ile)-lysidine synthetase protein
1066	S_1066	1	DNA ligase, NAD-dependent
1078	S_1078	0	Putative metalloprotease protein
1090	S_1090	0	Trigger factor
1091	S_1091	1	50S ribosomal protein L9

1094	S_1094	1	SsrA-binding protein
1097	S_1097	1	Chromosomal replication initiation protein (DnaA)
1099	S_1099	0	Radical SAM family enzyme
1113	S_1113	1	UDP-N-acetylenolpyruvoylglucosamine reductase
1116	S_1116	1	Ribosome-binding factor A
1119	S_1119	1	DNA primase
1127	S_1127	1	TRNA (guanine-N(1)-)-methyltransferase
1128	S_1128	0	30S ribosomal protein S20
1129	S_1129	1	Adenine-specific methyltransferase
1135	S_1135	1	Uroporphyrin-III C/tetrapyrrole methyltransferase
1137	S_1137	1	Transcription antitermination protein (NusB)
1148	S_1148	1	UDP-N-acetylmuramoyl-L-alanyl-D-glutamate synthetase
1149	S_1149	1	PriA; primosomal protein N'
1150	S_1150	1	DNA internalization-related competence protein (ComEC/Rec2)
1156	S_1156	1	Holliday junction DNA helicase (RuvB)
1159	S_1159	1	Iojap-like protein
1160	S_1160	1	Undecaprenyldiphospho-muramoylpentapeptide beta-N-acetylglucosaminyltransferase
1161	S_1161	1	Phosphopantetheine adenylyltransferase
1168	S_1168	1	Holliday junction DNA helicase (RuvA)
1173	S_1173	0	16S rRNA methyltransferase (GidB)
1181	S_1181	0	Putative ATPase or kinase UPF0079
1183	S_1183	0	Transcription-repair coupling factor
1193	S_1193	1	Holliday junction resolvase-like protein
1199	S_1199	1	50S ribosomal protein L25/general stress protein Ctc
1201	S_1201	1	M22 family glycoprotease peptidase
1203	S_1203	1	Recombination protein (RecR)
1204	S_1204	0	16S rRNA-processing protein (RimM)
1270	S_1270	0	DNA repair protein (RecN)
2094	S_2094	1	Threonyl-tRNA synthetase
4219	S_4219	2	Site-specific recombinase, phage integrase family protein
5357	S_5357	1	Endonuclease III
7219	S_7219	0	Ribosomal subunit pseudouridine synthase

C: cluster, S: singleton
