# Supplementary material for: A Deeply Branching Thermophilic Bacterium with an Ancient Acetyl-CoA Pathway Dominates a Subsurface Ecosystem
Source: PLoS One. 2012 Jan 27;7(1):e30559. doi: 10.1371/journal.pone.0030559 (PMC3267732; doi:10.1371/journal.pone.0030559)
Supplement: Table S2 — List of the prokaryotic species used for phylograms in Fig. 2 . (PDF) [file pone.0030559.s008.pdf]

Table S2 List of the prokaryotic species used for phylograms in Fig. 2

ID	Phylum/Class	Specis	Genome ID	Accession Number
1	*Thermotogae*	*Fervidobacterium nodosum* Rt17-B1	fno	NC_009718
2		*Petrotoga mobilis* SJ95	pmo	NC_010003
3		*Thermosipho melanesiensis* BI429	tme	NC_009616
4		*Thermotoga lettingae* TMO	tle	NC_009828
5		*Thermotoga maritima* MSB8	tma	NC_000853
6		*Thermotoga petrophila* RKU-1	tpt	NC_009486
7	**Candidate division OP1**	*Candidus* 'Acertothermus autotrophicum'	atm	AP011800-03
8	*Deinococcus/Thermus*	*Deinococcus geothermalis* DSM 11300	dge	NC_008025
9		*Deinococcus radiodurans* R1	dra	NC_001263-64
10		*Thermus thermophilus* HB27	tth	NC_005835
11	*Spirochaetes*	*Borrelia afzelii* PKo	baf	NC_008277
12		*Borrelia burgdorferi* B31	bbu	NC_001318
13		*Borrelia garinii* PBi	bga	NC_006156
14		*Leptospira borgpetersenii* serovar Hardjo-bovis JB197	lbj	NC_008510-11
15		*Treponema denticola* ATCC 35405	tde	NC_002967
16		*Treponema pallidumsub* sp. *pallidum* str. Nichols	tpa	NC_000919
17		*Leptospira interrogans* serovar Lai str. 56601	lil	NC_004342-43
18	*Chlorobi*	*Chlorobium chlorochromatii* CaD3	cch	NC_007514
19		*Chlorobium phaeobacteroides* DSM 266	cph	NC_008639
20		*Chlorobium tepidum* TLS	cte	NC_002932
21		*Pelodictyon luteolum* DSM 273	plt	NC_007512
22		*Prosthecochloris vibrioformis* DSM 265	pvi	NC_009337
23	*Bacteroidetes*	*Cytophaga hutchinsonii* ATCC 33406	chu	NC_008255
24		*Flavobacterium johnsoniae* UW101	fjo	NC_009441
25		*Gramella forsetii* KT0803	gfo	NC_008571
26		*Porphyromonas gingivalis* W83	pgi	NC_002950
27		*Salinibacter ruber* DSM 13855	sru	NC_007677
28		*Bacteroides fragilis* YCH46	bfr	NC_006347
29		*Bacteroides thetaiotaomicron* VPI-5482	bth	NC_004663
30	*Planctomycetes*	*Rhodopirellula baltica* SH 1	rba	NC_005027
31	*Chlamydiae*	Candidatus *Protochlamydia amoebophila* UWE25	pcu	NC_005861
32		*Chlamydia muridarum* Nigg	cmu	NC_002620
33		*Chlamydia trachomatis* D/UW-3/CX	ctr	NC_000117
34		*Chlamydophila abortus* S26/3	cab	NC_004552
35		*Chlamydophila caviae* GPIC	cca	NC_003361
36		*Chlamydophila pneumoniae* CWL029	cpn	NC_000922
37	*Aquificae*	*Aquifex aeolicus* VF5	aae	NC_000918
38	*ε-proteobacteria*	*Arcobacter butzleri* RM4018	abu	NC_009850
39		*Campylobacter fetus* subsp. fetus 82-40	cff	NC_008599
40		*Campylobacter jejuni* subsp. jejuni NCTC 11168	cje	NC_002163
41		*Helicobacter hepaticus* ATCC 51449	hhe	NC_004917
42		*Helicobacter pylori* 26695	hpy	NC_000915
43		*Sulfurimonas denitrificans* DSM 1251	tdn	NC_007575
44		*Sulfurovum* sp. NBC37-1	sun	NC_009663
45		*Wolinella succinogenes* DSM 1740	wsu	NC_005090
46		*Nitratiruptor* sp. SB155-2	nis	NC_009662
47	*δ-proteobacteria*	*Anaeromyxobacter* sp. Fw109-5	afw	NC_009675
48		*Bdellovibrio bacteriovorus* HD100	bba	NC_005363
49		*Desulfococcus oleovorans* Hxd3	dol	NC_009943
50		*Desulfotalea psychrophila* LSv54	dps	NC_006138
51		*Desulfovibrio desulfuricans* G20	dde	NC_007519
52		*Desulfovibrio vulgaris* subsp. *vulgaris* str. Hildenborough	dvu	NC_002937
53		*Geobacter metallireducens* GS-15	gme	NC_007517
54		*Geobacter sulfurreducens* PCA	gsu	NC_002939
55		*Geobacter uraniireducens* Rf4	gur	NC_009483
56		*Pelobacter carbinolicus* DSM 2380	pca	NC_007498

57		*Pelobacter propionicus* DSM 2379	ppd	NC_008609
58		*Sorangium cellulosum* 'So ce 56'	scl	NC_010162
59		*Syntrophobacter fumaroxidans* MPOB	sfu	NC_008554
60		*Syntrophus aciditrophicus* SB	sat	NC_007759
61		*Myxococcus xanthus* DK 1622	mxa	NC_008095
62	Unclassified proteobacteria	*Magnetococcus* sp. MC-1	mgm	NC_008576
63	*α-proteobacteria*	*Bradyrhizobium japonicum* USDA110	bja	NC_004463
64		*Bradyrhizobium* sp. ORS278	bra	NC_009445
65		*Brucella abortus biovar* 1 str. 9-941	bmb	NC_006932-33
66		*Brucella canis* ATCC 23365	bcs	NC_010103-104
67		*Brucella melitensis* 16M	bme	NC_003317-18
68		*Brucella ovis* ATCC 25840	bov	NC_009504-05
69		*Brucella suis* 1330	bms	NC_004310-11
70		Candidatus *Pelagibacter ubique* HTCC1062	pub	NC_007205
71		*Caulobacter crescentus* CB15	ccr	NC_002696
72		*Dinoroseobacter shibae* DFL 12	dsh	NC_009952
73		*Erythrobacter litoralis* HTCC2594	eli	NC_007722
74		*Gluconacetobacter diazotrophicus* PAl 5	gdi	NC_010125
75		*Gluconobacter oxydans* 621H	gox	NC_006677
76		*Granulobacter bethesdensis* CGDNIH1	gbe	NC_008343
77		*Hyphomonas neptunium* ATCC 15444	hne	NC_008358
78		*Jannaschia* sp. CCS1	jan	NC_007802
79		*Ochrobactrum anthropi* ATCC 49188	oan	NC_009667-68
80		*Parvibaculum lavamentivorans* DS-1	pla	NC_009719
81		*Rhizobium etli* CFN 42	ret	NC_007761
82		*Rhizobium leguminosarum* bv.*viciae* 3841	rle	NC_008380
83		*Rhodobacter sphaeroides* 2.4.1	rsp	NC_007493-94
84		*Rhodopseudomonas palustris* CGA009	rpa	NC_005296
85		*Rhodospirillum rubrum* ATCC 11170	rru	NC_007643
86		*Roseobacter denitrificans* OCh 114	rde	NC_008209
87		*Rubrobacter xylanophilus* DSM 9941	rxy	NC_008148
88		*Silicibacter pomeroyi* DSS-3	sil	NC_003911
89		*Silicibacter* sp. TM1040	sit	NC_008044
90		*Sinorhizobium medicae* WSM419	smd	NC_009636
91		*Sinorhizobium meliloti* 1021	sme	NC_003047
92		*Sphingomonas wittichii* RW1	swi	NC_009511
93		*Sphingopyxis alaskensis* RB2256	sal	NC_008048
94		*Wolbachia* endosymbiont of *Drosophila melanogaster*	wol	NC_002978
95		*Wolbachia* endosymbiont strain TRS of *Brugia malayi*	wbm	NC_006833
96		*Xanthobacter autotrophicus* Py2	xau	NC_009720
97		*Zymomonas mobilis* subsp. *mobilis* ZM4	zmo	NC_006526
98		*Magnetospirillum magneticum* AMB-1	mag	NC_007626
99		*Maricaulis maris* MCS10	mmr	NC_008347
100		*Mesorhizobium loti* MAFF303099	mlo	NC_002678
101		*Mesorhizobium* sp. BNC1	mes	NC_008254
102		*Methylobacterium extorquens* PA1	mex	NC_010172
103		*Neorickettsia sennetsu* str. Miyayama	nse	NC_007798
104		*Nitrobacter hamburgensis* X14	nha	NC_007964
105		*Nitrobacter winogradskyi* Nb-255	nwi	NC_007406
106		*Acidiphilium cryptum* JF-5	acr	NC_009484
107		*Agrobacterium tumefaciens* str. C58	atu	NC_003062-63
108		*Anaplasma marginale* str. St. Maries	ama	NC_004842
109		*Anaplasma phagocytophilum* HZ	aph	NC_007797
110		*Azorhizobium caulinodans* ORS 571	azc	NC_009937
111		*Bartonella bacilliformis* KC583	bbk	NC_008783
112		*Bartonella henselae* str. Houston-1	bhe	NC_005956
113		*Bartonella quintana* str. Toulouse	bqu	NC_005955
114		*Bartonella tribocorum* CIP 105476	btr	NC_010161

115	*β-proteobacteria*	*Acidovorax avenae* subsp. *citrulli* AAC00-1	aav	NC_008752
116		*Acidovorax* sp. JS42	ajs	NC_008782
117		*Azoarcus* sp. BH72	azo	NC_008702
118		*Azoarcus* sp. EbN1	eba	NC_006513
119		Bordetella bronchiseptica RB50	bbr	NC_002927
120		Bordetella parapertussis 12822	bpa	NC_002928
121		Bordetella pertussis Tohama I	bpe	NC_002929
122		*Bordetella petrii* DSM 12804	bpt	NC_010170
123		*Burkholderia ambifaria* AMMD	bac	NC_010551-52, 57
124		*Burkholderia cenocepacia* AU 1054	bcn	NC_008060-62
125		*Burkholderia multivorans* ATCC 17616	bmu	NC_010086-87. 84
126		*Burkholderia pseudomallei* K96243	bps	NC_006350-51
127		*Burkholderia* sp. 383	bur	NC_007509-11
128		*Burkholderia thailandensis* E264	bte	NC_007650-51
129		*Burkholderia vietnamiensis* G4	bvi	NC_009254-56
130		*Burkholderia xenovorans* LB400	bxe	NC_007951-53
131		*Chromobacterium violaceum* ATCC 12472	cvi	NC_005085
132		*Dechloromonas aromatica* RCB	dar	NC_007298
133		*Delftia acidovorans* SPH-1	dac	NC_010002
134		*Nitrosomonas europaea* ATCC 19718	neu	NC_004757
135		*Nitrosomonas eutropha* C91	net	NC_008344
136		*Nitrosospira multiformis* ATCC 25196	nmu	NC_007614
137		*Polaromonas* sp. JS666	pol	NC_007948
138		*Polynucleobacter* sp. QLW-P1DMWA-1	pnu	NC_009379
139		*Ralstonia solanacearum* GMI1000	rso	NC_003295
140		*Rhodoferax ferrireducens* T118	rfr	NC_007908
141		*Thiobacillus denitrificans* ATCC 25259	tbd	NC_007404
142		*Verminephrobacter eiseniae* EF01-2	vei	NC_008786
143		*Methylobacillus flagellatus* KT	mfa	NC_007947
144		*Neisseria gonorrhoeae* FA 1090	ngo	NC_002946
145		*Neisseria meningitidis* MC58	ngo	NC_002946
146	*γ-proteobacteria*	*Acinetobacter* sp. ADP1	aci	NC_005966
147		*Actinobacillus pleuropneumoniae* L20	apl	NC_009053
148		*Actinobacillus succinogenes* 130Z	asu	NC_009655
149		*Aeromonas hydrophila* subsp. *hydrophila* ATCC 7966	aha	NC_008570
150		*Aeromonas salmonicida* subsp. *salmonicida* A449	asa	NC_009348
151		*Alcanivorax borkumensis* SK2	abo	NC_008260
152		*Alkalilimnicola ehrlichei* MLHE-1	aeh	NC_008340
153		*Baumannia cicadellinicola* str. Hc (*Homalodisca coagulata*)	bci	NC_007984
154		*Buchnera aphidicola* str. APS (*Acyrthosiphon pisum*)	buc	NC_002528
155		Candidatus *Blochmannia floridanus*	bfl	NC_005061
156		Candidatus *Blochmannia pennsylvanicus* str. BPEN	bpn	NC_007292
157		Candidatus *Ruthia magnifica* str. Cm (*Calyptogena magnifica*)	rma	NC_008610
158		Candidatus *Vesicomyosocius okutanii* HA	vok	NC_009465
159		*Chromohalobacter salexigens* DSM 3043	csa	NC_007963
160		*Citrobacter koseri* ATCC BAA-895	cko	NC_009792
161		*Colwellia psychrerythraea* 34H	cps	NC_003910
162		*Coxiella burnetii* RSA 493	cbu	NC_002971
163		*Ehrlichia canis* str. Jake	ecn	NC_007354
164		*Ehrlichia chaffeensis* str. Arkansas	ech	NC_007799
165		*Ehrlichia ruminantium* str. Welgevonden	eru	NC_005295
166		*Enterobacter sakazakii* ATCC BAA-894	esa	NC_009778
167		*Enterobacter* sp. 638	ent	NC_009436
168		*Erwinia carotovora* subsp. *atroseptica* SCRI1043	eca	NC_004547
169		*Escherichia coli* K-12 MG1655	eco	NC_000913
170		*Francisella tularensis* subsp. *novicida* U112	ftn	NC_008601
171		*Francisella tularensis* subsp. tularensis SCHU S4	ftu	NC_006570
172		*Haemophilus ducreyi* 35000HP	hdu	NC_002940

173		*Haemophilus influenzae* Rd KW20	hin	NC_000907
174		*Haemophilus somnus* 129PT	hso	NC_008309
175		*Hahella chejuensis* KCTC 2396	hch	NC_007645
176		*Halorhodospira halophila* SL1	hha	NC_008789
177		*Idiomarina loihiensis* L2TR	ilo	NC_006512
178		*Klebsiella pneumoniae* subsp. *pneumoniae* MGH 78578	kpn	NC_009648
179		*Legionella pneumophila* subsp. *pneumophila* str. Philadelphia 1	lpn	NC_002942
180		*Pasteurella multocida* subsp. *multocidastr*. Pm70	pmu	NC_002663
181		*Photobacterium profundum* SS9	ppr	NC_006370-71
182		*Photorhabdus luminescens* subsp. *laumondii* TTO1	plu	NC_005126
183		*Pseudoalteromonas atlantica* T6c	pat	NC_008228
184		*Pseudoalteromonas haloplanktis* TAC125	pha	NC_007481-82
185		*Pseudomonas aeruginosa* PAO1	pae	NC_002516
186		*Pseudomonas entomophila* L48	pen	NC_008027
187		*Pseudomonas fluorescens* Pf-5	pfl	NC_004129
188		*Pseudomonas mendocina* ymp	pmy	NC_009439
189		*Pseudomonas putida* KT2440	ppu	NC_002947
190		*Pseudomonas stutzeri* A1501	psa	NC_009434
191		*Pseudomonas syringae* pv. *phaseolicola* 1448A	psp	NC_005773
192		*Pseudomonas syringae* pv. *syringae* B728a	psb	NC_007005
193		*Pseudomonas syringae* pv. *tomato* str. DC3000	pst	NC_004578
194		*Psychrobacter arcticus* 273-4	par	NC_007204
195		*Psychrobacter cryohalolentis* K5	pcr	NC_007969
196		*Psychrobacter* sp. PRwf-1	prw	NC_009524
197		*Psychromonas ingrahamii* 37	pin	NC_008709
198		*Saccharophagus degradans* 2-40	sde	NC_007912
199		*Salmonella typhimurium* LT2	stm	NC_003197
200		*Serratia proteamaculans* 568	spe	NC_009832
201		*Shewanella amazonensis* SB2B	saz	NC_008700
202		*Shewanella denitrificans* OS217	sdn	NC_007954
203		*Shewanella frigidimarina* NCIMB 400	sfr	NC_008345
204		*Shewanella loihica* PV-4	slo	NC_009092
205		*Shewanella oneidensis* MR-1	son	NC_004347
206		*Shewanella pealeana* ATCC 700345	spl	NC_009901
207		*Shewanella putrefaciens* CN-32	spc	NC_009438
208		*Shewanella sediminis* HAW-EB3	sse	NC_009831
209		*Shewanella* sp. ANA-3	shn	NC_008577
210		*Shewanella* sp. MR-4	she	NC_008321
211		*Shewanella* sp. MR-7	shm	NC_008322
212		*Shewanella* sp. W3-18-1	shw	NC_008750
213		*Shigella boydii* Sb227	sbo	NC_007613
214		*Shigella dysenteriae* Sd197	sdy	NC_007606
215		*Shigella flexneri* 2a str. 301	sfl	NC_004337
216		*Shigella sonnei* Ss046	ssn	NC_007384
217		*Thiomicrospira crunogena* XCL-2	tcx	NC_007520
218		*Vibrio cholerae* O1 biovar eltor str. N16961	vch	NC_002505-06
219		*Vibrio fischeri* ES114	vfi	NC_006840-41
220		*Vibrio harveyi* ATCC BAA-1116	vha	NC_009783-84
221		*Vibrio vulnificus* CMCP6	vvu	NC_004459-60
222		*Wigglesworthia glossinidia* endosymbiont of *Glossina brevipalpis*	wbr	NC_004344
223		*Xanthomonas axonopodis* pv. citristr. 306	xac	NC_003919
224		*Xanthomonas campestris* pv. campestris str. ATCC 33913	xcb	NC_007086
225		*Xanthomonas campestris* pv. vesicatoria str. 85-10	xcv	NC_007508
226		*Xanthomonas oryzae* pv. *oryzae* KACC10331	xoo	NC_006834
227		*Xylella fastidiosa* 9a5c	xfa	NC_002488
228		*Yersinia enterocolitica* subsp. *enterocolitica* 8081	yen	NC_008800, NC_008791
229		*Yersinia pestis* CO92	ype	NC_003143
230		*Yersinia pseudotuberculosis* IP 32953	yps	NC_006155

231		*Mannheimia succiniciproducens* MBEL55E	msu	NC_006300
232		*Marinobacter aquaeolei* VT8	maq	NC_008740
233		*Marinomonas* sp. MWYL1	mmw	NC_009654
234		*Nitrosococcus oceani* ATCC 19707	noc	NC_007484
235	*Acidobacteria*	*Solibacter usitatus* Ellin6076	sus	NC_008536
236		*Acidobacteria bacterium* Ellin345	aba	NC_008009
237	*Tenericutes*	Onion yellows phytoplasma OY-M	poy	NC_005303
238		*Ureaplasma parvumserovar* 3 str. ATCC 700970	uur	NC_002162
239		*Acholeplasma laidlawii* PG-8A	acl	NC_010163
240		Aster yellows witches'-broom phytoplasma AYWB	ayw	NC_007716
241		*Mesoplasma florum* L1	mfl	NC_006055
242		*Mycoplasma agalactiae* PG2	maa	NC_009497
243		*Mycoplasma capricolum* subsp. *capricolum* ATCC 27343	mcp	NC_007633
244		*Mycoplasma gallisepticum* R	mga	NC_004829
245		*Mycoplasma hyopneumoniae* 232	mhy	NC_006360
246		*Mycoplasma mobile* 163K	mmo	NC_006908
247		*Mycoplasma mycoides* subsp. *mycoides* SC str. PG1	mmy	NC_005364
248		*Mycoplasma penetrans* HF-2	mpe	NC_004432
249	*Firmicutes*	*Caldicellulosiruptor saccharolyticus* DSM 8903	csc	NC_009437
250		*Carboxydothermus hydrogenoformans* Z-2901	chy	NC_007503
251		*Clostridium acetobutylicum* ATCC 824	cac	NC_003030
252		*Clostridium beijerinckii* NCIMB 8052	cbe	NC_009617
253		*Clostridium botulinum* A str. ATCC 3502	cbo	NC_009495
254		*Clostridium kluyveri* DSM 555	ckl	NC_009706
255		*Clostridium novyi* NT	cno	NC_008593
256		*Clostridium perfringens* str. 13	cpe	NC_003366
257		*Clostridium phytofermentans* ISDg	cpy	NC_010001
258		*Clostridium thermocellum* ATCC 27405	cth	NC_009012
259		*Desulfitobacterium hafniense* Y51	dsy	NC_007907
260		*Desulfotomaculum reducens* MI-1	drm	NC_009253
261		*Enterococcus faecalis* V583	efa	NC_004668
262		*Geobacillus kaustophilus* HTA426	gka	NC_006510
263		*Geobacillus thermodenitrificans* NG80-2	gtn	NC_009328
264		*Lactobacillus acidophilus* NCFM	lac	NC_006814
265		*Lactobacillus casei* ATCC 334	lca	NC_008526
266		*Lactobacillus delbrueckii* subsp. *bulgaricus* ATCC 11842	ldb	NC_008054
267		*Lactobacillus gasseri* ATCC 33323	lga	NC_008530
268		*Lactobacillus helveticus* DPC 4571	lhe	NC_010080
269		*Lactobacillus johnsonii* NCC 533	ljo	NC_005362
270		*Lactobacillus plantarum* WCFS1	lpl	NC_004567
271		*Lactobacillus sakei* subsp. Sakei 23K	lsa	NC_007576
272		*Lactobacillus salivarius* UCC118	lsl	NC_007929
273		*Lactococcus lactis* subsp. lactis IL1403	lla	NC_002662
274		*Oceanobacillus iheyensis* HTE831	oih	NC_004193
275		*Oenococcus oeni* PSU-1	ooe	NC_008528
276		*Pediococcus pentosaceus* ATCC 25745	ppe	NC_008525
277		*Pelotomaculum thermopropionicum* SI	pth	NC_009454
278		*Staphylococcus aureus* subsp. *aureus* N315	sau	NC_002745
279		*Staphylococcus epidermidis* ATCC 12228	sep	NC_004461
280		*Staphylococcus haemolyticus* JCSC1435	sha	NC_007168
281		*Staphylococcus saprophyticus* subsp. *saprophyticus* ATCC 15305	ssp	NC_007350
282		*Streptococcus agalactiae* 2603V/R	sag	NC_004116
283		*Streptococcus gordonii* str. Challis substr. CH1	sgo	NC_009785
284		*Streptococcus mutans* UA159	smc	NC_013928
285		*Streptococcus pneumoniae* TIGR4	spn	NC_003028
286		*Streptococcus pyogenes* M1 GAS	spy	NC_002737
287		*Streptococcus sanguinis* SK36	ssa	NC_009009
288		*Streptococcus thermophilus* CNRZ1066	stc	NC_006449

289		*Symbiobacterium thermophilum* IAM 14863	sth	NC_006177
290		*Syntrophomonas wolfei* subsp. wolfeistr. Goettingen	swo	NC_008346
291		*Thermoanaerobacter ethanolicus* ATCC 33223	tpd	NC_010321
292		*Thermoanaerobacter ethanolicus* X514	tex	NC_010320
293		*Thermoanaerobacter tengcongensis* MB4	tte	NC_003869
294		*Alkaliphilus metalliredigens* QYMF	amt	NC_009633
295		*Bacillus amyloliquefaciens* FZB42	bay	NC_009725
296		*Bacillus anthracis* str. Ames	ban	NC_003997
297		*Bacillus clausii* KSM-K16	bcl	NC_006582
298		*Bacillus licheniformis* ATCC 14580	bli	NC_006270
299		*Bacillus subtilis* subsp. subtilis str. 168	bsu	NC_000964
300		*Bacillus thuringiensis* serovar konkukian str. 97-27	btk	NC_005957
301		*Bacillus weihenstephanensis* KBAB4	bwe	NC_010184
302		*Listeria innocua* Clip11262	lin	NC_003212
303		*Listeria monocytogenes* EGD-e	lmo	NC_003210
304		*Listeria welshimeri* serovar 6b str. SLCC5334	lwe	NC_008555
305		*Moorella thermoacetica* ATCC 39073	mta	NC_007644
306	*Chloroflexi*	*Chloroflexus aurantiacus* J-10-fl	cau	NC_010175
307		*Dehalococcoides ethenogenes* 195	det	NC_002936
308		*Dehalococcoides* sp. BAV1	deb	NC_009455
309		*Dehalococcoides* sp. CBDB1	deh	NC_007356
310		*Herpetosiphon aurantiacus* ATCC 23779	hau	NC_009972
311		*Roseiflexus castenholzii* DSM 13941	rca	NC_009767
312		*Roseiflexus* sp. RS-1	rrs	NC_009523
313	*Cyanobacteria*	*Gloeobacter violaceus* PCC 7421	gvi	NC_005125
314		*Prochlorococcus marinus* subsp. *marinus* str. CCMP1375	pma	NC_005042
315		*Synechococcus elongatus* PCC 6301	syc	NC_006576
316		*Synechococcus* sp. CC9311	syg	NC_008319
317		*Synechococcus* sp. CC9605	syd	NC_007516
318		*Synechococcus* sp. CC9902	sye	NC_007513
319		*Synechococcus* sp. JA-2-3B'a(2-13)	cyb	NC_007776
320		*Synechococcus* sp. RCC307	syr	NC_009482
321		*Synechococcus* sp. WH 7803	syx	NC_009481
322		*Synechococcus* sp. WH 8102	syw	NC_005070
323		*Synechocystis* sp. PCC 6803	syn	NC_000911
324		*Thermosynechococcus elongatus* BP-1	tel	NC_004113
325		*Trichodesmium erythraeum* IMS101	ter	NC_008312
326	*Actinobacteria*	*Bifidobacterium adolescentis* ATCC 15703	bad	NC_008618
327		*Clavibacter michiganensis* subsp. *michiganensis* NCPPB 382	cmi	NC_009480
328		*Corynebacterium efficiens* YS-314	cef	NC_004369
329		*Corynebacterium glutamicum* ATCC 13032	cgl	NC_003450
330		*Corynebacterium jeikeium* K411	cjk	NC_007164
331		*Frankia alni* ACN14a	fal	NC_008278
332		*Frankia* sp. EAN1pec	fre	NC_009921
333		*Kineococcus radiotolerans* SRS30216	kra	NC_009664
334		*Leifsonia xyli* subsp. *xyli* str. CTCB07	lxx	NC_006087
335		*Propionibacterium acnes* KPA171202	pac	NC_006085
336		*Renibacterium salmoninarum* ATCC 33209	rsa	NC_010168
337		*Rhodococcus jostii* RHA1	rha	NC_008268
338		*Saccharopolyspora erythraea* NRRL 2338	sen	NC_009142
339		*Salinispora arenicola* CNS-205	saq	NC_009953
340		*Salinispora tropica* CNB-440	stp	NC_009380
341		*Streptococcus suis* 05ZYH33	ssu	NC_009442
342		*Streptomyces avermitilis* MA-4680	sma	NC_003155
343		*Streptomyces coelicolor* A3(2)	sco	NC_003888
344		*Thermobifida fusca* YX	tfu	NC_007333
345		*Tropheryma whipplei* TW08/27	tws	NC_004551
346		*Acidothermus cellulolyticus* 11B	ace	NC_008578

347		*Arthrobacter* sp. FB24	art	NC_008541
348		*Mycobacterium avium* subsp. *paratuberculosis* K-10	mpa	NC_002944
349		*Mycobacterium bovis* AF2122/97	mbo	NC_002945
350		*Mycobacterium gilvum* PYR-GCK	mgi	NC_009338
351		*Mycobacterium leprae* TN	mle	NC_002677
352		*Mycobacterium smegmatis* str. MC2 155	msm	NC_008596
353		*Mycobacterium* sp. JLS	mjl	NC_009077
354		*Mycobacterium* sp. KMS	mkm	NC_008705
355		*Mycobacterium* sp. MCS	mmc	NC_008146
356		*Mycobacterium tuberculosis* H37Rv	mtu	NC_000962
357		*Mycobacterium ulcerans* Agy99	mul	NC_008611
358		*Mycobacterium vanbaalenii* PYR-1	mva	NC_008726
359	*Crenarchaeota*	*Pyrobaculum aerophilum* IM2	pai	NC_003364
360		*Sulfolobus solfataricus* P2	sso	NC_002754
361		*Aeropyrum pernix* K1	ape	NC_000854
362	*Euryarchaeota*	*Halobacterium salinarium* NRC-1	hal	NC_002607
363		*Pyrococcus horikoshii* OT3	pho	NC_000961
364		*Thermoplasma acidophilum* DSM 1728	tac	NC_002578
365		*Archaeoglobus fulgidus* DSM 4304	afu	NC_000917
366		*Methanococcus jannaschii* DSM 2661	mja	NC_000909
367	*Thaumarchaeota*	*Nitrosopumilus maritimus* SCM1	nmr	NC_010085
