# Supplementary material for: A Deeply Branching Thermophilic Bacterium with an Ancient Acetyl-CoA Pathway Dominates a Subsurface Ecosystem
Source: PLoS One. 2012 Jan 27;7(1):e30559. doi: 10.1371/journal.pone.0030559 (PMC3267732; doi:10.1371/journal.pone.0030559)
Supplement: Table S3 — List of species or clone name used for phylogenetic analysis based on 16S rRNA genes. (PDF) [file pone.0030559.s009.pdf]

Table S3 List of species or clone name used for phylogenetic analysis based on 16S rRNA genes

Domain	Phylum	Species/Clone	Acsession nos.
Archaea	Crenarchaeota	*Sulfolobus acidocaldarius*	D14876
	Euryarchaeota	*Methanococcus vannielii*	M36507
Bacteria	Acidobacteria	Uncultured *Holophaga/Acidobacterium* Sva0450	AJ240998
		Unidentified bacterium clone NKB17	AB013269
		Uncultured *Acidobacteria* bacterium clone TDNP_Wbc97_51_2_207	FJ517114
	Actinobacteria	*Acidimicrobium ferrooxidans*	U75647
		Ferrimicrobium acidiphilum strain T23	AF251436
		Acidimicrobium ferrooxidans DSM 10331	NC_013124
	Aquificae	*Desulfurobacterium thermolithotrophum*	AJ001049
		Uncultured bacterium VC2.1 Bac48	AF068811
		Unidentified *Aquificales* OPB13	AF027098
	Bacteroidetes	*Thermonema lapsumsequence*	L11703
		Bacteroides fragilis NCTC 9343	NC_003228
		Bacteroides thetaiotaomicron VPI-5482	NC_004663
	Caldiserica	*Filamentous bacterium* AZM16c01	AB428365
		Uncultured bacterium SRI-280	AF255600
		Uncultured *Caldiserica* bacterium clone NRB39	HM041956
	**Candidate division OP1**	Uncultured bacterium 'KTK 27'	AJ133616
		Uncultured bacterium 'KTK 41'	AJ133619
		Candidus 'Acertothermus autotrophicum'	AP011701
	Candidate division OP10	Uncultured bacterium SBR1039	X84482
		Candidate division OP10 clone OPB80	AF027089
		Uncultured candidate division OP10 bacterium clone SBRT161	AF368187
	Candidate division OP11	Uncultured eubacterium WCHB1-64	AF050606
		Uncultured eubacterium WCHB1-26	AF050599
		Candidate division OP11 clone OPB92	AF027030
	Candidate division OP2	Unidentified *Cytophagales* OPB88	AF027006
		Uncultured bacterium clone R15	AF407687
		Uncultured bacterium clone Y63	AF407680
	Candidate division OP3	Unclassified bacterial species isolate koll11	AJ224540
		Unidentified bacterium gene for clone: BD3-9	AB015551
		Unidentified bacterium gene for clone: BD4-9	AB015559
	Candidate division OP8	Candidate division OP8 clone OPB5	AF027067
		Unidentified bacterium clone: NKB18	AB013270
		Uncultured bacterium clone: ODP1230B20.28	AB177172
	Candidate division OP9	Benzene mineralizing consortium clone SB-45	AF029050
		Uncultured candidate division JS1 bacterium clone JTB138	AB015269
		Uncultured bacteriumclone: N16	AB195913
	Chlamydiae	*Chlamydia trachomatis* D/UW-3/CX	NC_000117
		Chlamydia muridarum Nigg	NC_002620
		Chlamydia trachomatis A/HAR-13	NC_007429
	Chlorobi	Unidentified Cytophagales/green sulfur bacterium OPB56	AF027009
		Uncultured bacterium clone BIfdi48	AJ318130
		Uncultured bacterium clone RB029	AB240289
	Chloroflexi	Unidentified green non-sulfur bacterium OPB11	AF027032
		Unidentified bacterium	Z94009
		Chloroflexus aurantiacus J-10-fl	NC_010175
	Chrysiogenetes	*Chrysiogenes arsenatis*	X81319
		Dissimilatory selenate-respiring bacterium S5	DQ991965
		Bacterium AHT 19	GQ922843

	Cyanobacteria	*Prochlorococcus marinus* subsp. pastoris	AF180967
		Leptolyngbya foveolarum	X84808
		Acaryochloris marina MBIC11017	NC_009925
	Deferribacteres	Bacterium ASF457	AF157055
		Denitrovibrio acetophilus	AF146526
		Deferribacter desulfuricans SSM1	NC_013939
	Deinococcus-Thermus	*Deinococcus geothermalis* strain E50053	AJ000002
		Deinococcus radiodurans	M21413
		Thermus aquaticus YT-1	L09663
	Dictyoglomi	*Dictyoglomus turgidum* DSM 6724	NC_011661
		Dictyoglomus sp. 1512	FJ626840
		Dictyoglomus sp. TC38	HM004611
	Fibrobacteres	*Fibrobacter succinogenes* subsp. elongatus strain HM2	GU269553
		Fibrobacter succinogenes subsp. succinogenes S85	NC_013410
		Uncultured *Fibrobacterales* bacterium clone 290cost002-P3L-969	EF455006
	Firmicutes	*Paenibacillus polymyxa* strain: IAM 13419	D16276
		Peptococcus niger	X55797
		Alicyclobacillus acidiphilus strain TA-67	NR_028637
	Fusobacteria	*Streptobacillus moniliformis* (ATCC 14647 Type strain)	Z35305
		Cetobacterium ceti strain M-3333; NCFB 3026	X78419
		Sebaldella termitidis ATCC 33386	NC_013517
	Gemmatimonadetes	Uncultured bacterium SBRH63	AF268993
		Gemmatimonas aurantiaca T-27	NC_012489
		Gemmatimonas sp. enrichment culture clone AOCRB-EC-6	GU557153
	Lentisphaerae	Unidentified eubacterium clone vadinHB65	U81755
		Unidentified eubacterium clone vadinBE97	U81707
		Victivallaceae bacterium NML 080035	FJ394915
	Nitrospirae	*Magnetobacterium bavaricum*	X71838
		Leptospirillum sp. NOen1	AF376016
		Candidatus *Nitrospira defluvii* clone B14	GQ249372
	Planctomycetes	Uncultured eubacterium WD283	AJ292685
		Unidentified eubacterium clone vadinHA49	U81766
		Pirellula staleyi DSM 6068	NC_013720
	Proteobacteria	*Rhodopseudomonas palustris* BisB5	CP000283
		Burkholderia mallei ATCC 23344	CP000011
		Pseudomonas aeruginosa PAO1	AE004091
	Spirochaetes	*Brachyspira hyodysenteriae* strain B78(rrn) gene	U14930
		Candidate division OP4 clone OPB40	AF027047
		Brachyspira hyodysenteriae WA1	NC_012225
	Synergistetes	Unidentified eubacterium clone vadinBB02	U81658
		Unidentified eubacterium clone vadinHA73	U81735
		Uncultured bacterium BB48	AF129869
	Thermodesulfobacteria	Unidentified *Thermodesulfobacterium* group OPB45	AF027096
		Thermodesulfotobacterium sp. SRI-93	AF255596
		Caldimicrobium rimae strain DS	EF554596
	Thermotogae	*Petrotoga mobilis*	Y15479
		Thermosipho sp. DSM 13256	AJ272022
		Thermotoga maritima	M21774
	Verrucomicrobia	Uncultured bacterium clone DA101	Y07576
		Opitutus terrae PB90-1	NC_010571
		Coraliomargarita akajimensis DSM 45221	NC_014008
